# Engineering strategies and binding mechanisms of therapeutic anti–PD-1 antibodies approved by regulatory agencies globally

**DOI:** 10.3389/fimmu.2026.1834585

**Published:** 2026-05-21

**Authors:** Juan C. Almagro, Keyla Gómez-Castellano, Ileana Licona-Limón, Gregorio de Jesús Carballo Uicab, Frida D. Ramírez-Villedas, Alejandra Montes-Luna, Edith González-González, Silvia Godínez-Palma, Hugo A. Barrera Saldaña, Sonia M. Pérez-Tapia

**Affiliations:** 1 GlobalBio, Inc., Cambridge, MA, United States; 2 Unidad de Desarrollo e Investigación en Bioterapéuticos (UDIBI), Escuela Nacional de Ciencias Biológicas, Instituto Politécnico Nacional, Mexico City, Mexico; 3 Laboratorio Nacional para Servicios Especializados de Investigación, Desarrollo e Innovación (I+D+I) para Farmoquímicos y Biotecnológicos, LANSEIDI-FarBiotec-CONACyT, Mexico City, Mexico; 4 Dirección de Investigación Científica Básica, Laboratorios Columbia SA de CV, Mexico City, Mexico; 5 Departamento de Inmunología, Escuela Nacional de Ciencias Biológicas, Instituto Politécnico Nacional (ENCB-IPN), Mexico City, Mexico

**Keywords:** antibody engineering, bispecific antibodies, cancer immunotherapy, immune checkpoint blockade, PD-1:PD-L1 interaction, programmed cell death protein 1

## Abstract

Anti-PD-1 therapeutic antibodies have represented a paradigm-shift in cancer treatment. The success of the first two anti-PD-1 antibodies approved by the FDA in 2014, pembrolizumab and nivolumab, has spurred an explosion in the discovery, engineering and development of anti-PD-1 antibody-based drugs. Currently, 16 monospecific and two bispecific anti-PD-1 therapeutic antibodies have been approved by regulatory agencies in the United States, Europe, China, and Japan. In this review, we compiled and discussed the available data on these 18 therapeutic anti-PD-1-based drugs. We first summarized the PD-1 signaling pathway and tumor immune escape mechanisms. Second, we discussed the structure of PD-1 and its molecular interaction with PD-L1/L2. Third, we reviewed the antibody engineering strategies used to optimize the variable (V) and Fc regions of the 18 approved antibodies. Fourth, we compared the affinity for PD-1 and PD-1:PD-L1 blockade activity, as well as reviewed the mechanism of binding of the published structures of PD-1 in complex with the antibodies. In doing so, we highlighted commonalities and differences of the interplay between affinity, blocking of the PD-1:PD-L1 interaction and regions recognized on PD-1 that contributed to the functional performance of the anti–PD-1 antibodies approved by regulatory agencies globally to treat cancer.

## 1 Introduction

Therapeutic antibodies have revolutionized cancer treatment, with over 100 antibody-based drugs approved worldwide for oncology indications as of April 2026 (1). Early therapeutic antibodies targeted tumor-associated antigens and eliminated cancer cells through immune effector mechanisms such as antibody-dependent cellular cytotoxicity (ADCC), antibody-dependent cellular phagocytosis (ADCP), and complement-dependent cytotoxicity (CDC) (2–4), or by interfering with signaling pathways involved in tumor growth (5). In 2018, James P. Allison and Tasuku Honjo were awarded the Nobel Prize in Physiology or Medicine for their discovery of cancer therapies based on the inhibition of negative immune regulation (6). This groundbreaking work established a paradigm-shift approach to cancer therapy by restoring T-cell activity rather than binding a specific molecule or receptor in the cancer cells and inducing ADCC, CDC and/or ADCP (6).

Allison’s seminal work in the early 1990s focused on CTLA-4 (cytotoxic T-lymphocyte-associated protein 4). CTLA-4 is expressed on T cells and functions as a negative regulator of the immune response, acting as an immune checkpoint (7, 8). CTLA-4 blockade prevents its interaction with B7 ligands (CD80/CD86), shifts the balance toward CD28-mediated co-stimulation, unleashes T-cell activation and enhances antitumor immune responses (7, 9). In parallel, Honjo demonstrated that antibody-mediated blockade of PD-1 (Programmed cell death protein 1, CD279), another immune checkpoint, and its ligands PD-L1 and PD-L2 (Programmed cell death ligands 1 and 2) also known as CD274 and CD273, respectively, produced stronger antitumor effects than CTLA-4 inhibition, highlighting the therapeutic potential of targeting PD-1 across diverse malignancies (10, 11).

Building upon Allison’s pioneering work, in 2011 the fully human anti-CTLA-4 monoclonal antibody ipilimumab (commercial name Yervoy^®^) became the first immune checkpoint inhibitor approved by the U.S. Food and Drug Administration (FDA) for the treatment of metastatic melanoma (12). In September 2014, based on Honjo’s pioneering work, the FDA granted accelerated approval of pembrolizumab (Keytruda^®^), the first anti-PD-1 therapeutic antibody to treat patients with unresectable or metastatic melanoma following progression on prior therapy (13). A few months later, in December 2014, the FDA approved the second anti-PD-1 antibody, nivolumab (Opdivo^®^), for the treatment of unresectable or metastatic melanoma (14). Pembrolizumab and nivolumab were further approved for cancer therapy by the European Medicines Agency (EMA), the Japanese Pharmaceuticals and Medical Devices Agency (PMDA) and the Chinese National Medical Products Administration (NMPA).

**Figure 1:**
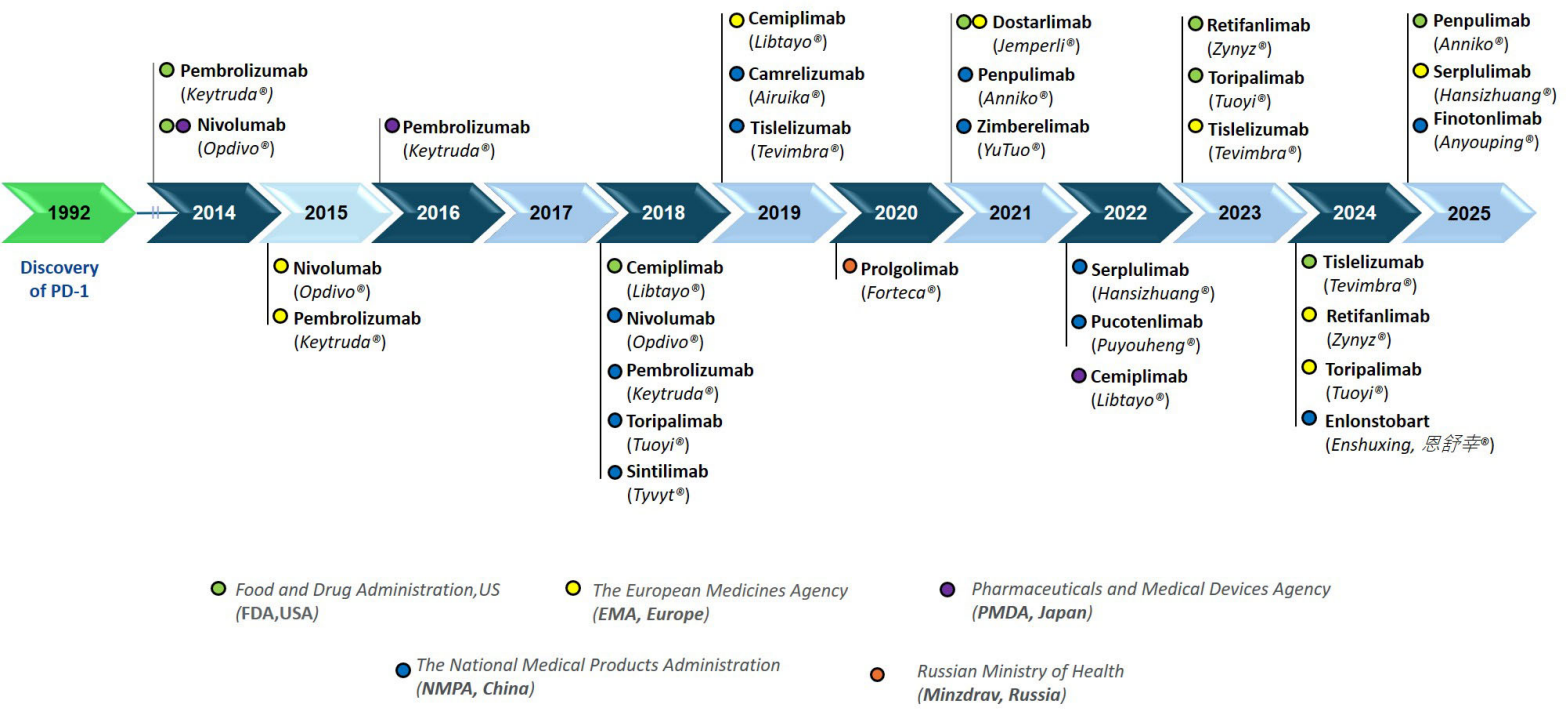
Timeline of anti-PD-1 therapeutic antibodies approvals by regulatory agencies worldwide. The data was sourced from The Antibody Society (1) and the Immunogenetics database (IMGT) (15), and confirmed with the information published by the regulatory agencies that approved their clinical use.

The therapeutic success of these two anti-PD-1 antibodies spurred an explosion in the discovery, engineering and development of new anti-PD-1 therapeutic antibodies over the past decade (**Figure 1**). The FDA and EMA approved cemiplimab (Libtayo^®^), dostarlimab (Jemperli^®^), retifanlimab (Zynyz^®^), toripalimab (Loqtorzi^®^) and tislelizumab (Tevimbra^®^). More recently, penpulimab (ANNIKO^®^) was approved by the FDA and serplulimab (Hetronifly^®^/HANSIZHUANG^®^) received approval from the EMA. In parallel with FDA and/or EMA approvals, NMPA has approved multiple anti-PD-1 antibodies including sintilimab (Tyvyt^®^), camrelizumab (AiRuiKa^®^), zimberelimab (YuTuo^®^), pucotenlimab (Puyouheng^®^), enlonstobart (Enshuxing, 恩舒幸^®^), and finotonlimab (Anyouping, 安佑平^®^). Meanwhile, the Ministry of Health of the Russian Federation (Minzdrav) approved prolgolimab (Forteca^®^); amounting to 16 approved anti-PD-1 therapeutic antibodies to treat cancer globally.

Beyond the approval of anti-PD-1 monospecific antibodies, an innovative generation of biotherapeutics combining anti-PD-1 antibodies or fragments with modules that bind other targets have recently been approved or are in phase 2/3 or 3 of clinical investigation. Cadonilimab (开坦尼^®^ - Kaitanni), a bispecific PD-1/CTLA-4 antibody, received NMPA approval in 2022 (16). Ivonescimab (依达方 - Yidafang), combining an anti-PD-1 module with bevacizumab was approved by NMPA in 2024 and is currently in Phase 3 clinical trials in US (17). Bevacizumab inhibits the vascular endothelial growth factor A (VEGF-A) (18), a key cytokine that stimulates angiogenesis. Additional bispecific molecules linking anti-PD-1 antibodies or fragments with diverse modules targeting oncology relevant molecules are in late stages of clinical trials and are expected to reach approval for clinical use in the near future (19).

In this review, we compiled and discussed the available data on the 16 globally approved monospecific anti-PD-1 therapeutic antibodies (**Table 1**), with focus on the engineering methods to optimize these molecules for therapeutic settings. We first described the PD-1 signaling pathway, tumor immune escape mechanisms and the structure of PD-1 and its molecular interaction with PD-L1/L2 to provide biological context for the discussion of the antibody mechanisms of binding to PD-1. Second, we discussed the engineering strategies used to optimize the antibody Variable (V) regions and crystallizable fragment (Fc) of these anti-PD-1-based drugs. Third, we compared the affinity of the antibodies for human PD-1 (hPD-1), hPD-1:PD-L1 *in vitro* blockade, and the epitope recognized on hPD-1. Finally, we summarized the current information on the two approved anti-PD-1-based bispecific molecules.

**Table 1:** Approved monospecific anti-PD-1 therapeutic antibodies.

#	INN*	Commercial name	Company	V regions	Isotype	Source of V regions	First reviewed/approved indication
1	Pembrolizumab	Keytruda^®^	Merck and Co.	Humanized	IgG4 S228P	Murine hybridoma–derived antibody humanized by CDR grafting	Melanoma
2	Nivolumab	Opdivo^®^	Bristol-Myers Squibb	Human	IgG4 S228P	Medarex HuMAb^®^ transgenic mouse	Melanoma, non-small cell lung cancer
3	Toripalimab	LOQTORZI^®^, Tuoyi^®^	Shanghai Junshi Biosciences Co., Ltd. (Junshi Biosciences), Coherus BioSciences	Humanized	IgG4 S228P	Hybridoma-derived parental antibody; humanized by computational design and phage display	Unresectable or metastatic melanoma
4	Cemiplimab	Libtayo^®^	Regeneron Pharmaceuticals, Inc./Sanofi	Human	IgG4 S228P	Transgenic mouse (Regeneron VelocImmune^®^ platform)	Cutaneous squamous cell carcinoma
5	Sintilimab	Tyvyt^®^	Innovent Biologics, Inc and Eli Lilly & Company	Human	IgG4 S228P	Yeast display technology	Hodgkin’s lymphoma
6	Camrelizumab	AiRuiKa^®^	Jiangsu HengRui Medicine Co., Ltd.	Humanized	IgG4 S228P	Murine hybridoma Mab005	Hodgkin’s lymphoma
7	Tislelizumab	Tevimbra^®^/Baizean^®^	Novartis and BeiGene, Ltd	Humanized	IgG4 S228P/E233P/F234V/L235A/D265A	Murine hybridoma	Esophageal squamous cell carcinoma
8	Prolgolimab	Forteca^®^	BIOCAD	Human	IgG1 L234A/L235A	Phage display	Unresectable or metastatic melanoma
9	Dostarlimab	Jemperli^®^	GSK plc/AnaptysBio/TESARO, Inc	Humanized	IgG4 S228P	Mouse hybridoma	Endometrial cancer
10	Penpulimab	ANNIKO^®^	Akeso, Inc. (Akeso Biopharma)/Sino Biopharmaceutical Limited	Humanized	IgG1 L234A L235A G237A	mouse hybridoma	Metastatic nasopharyngeal carcinoma
11	Zimberelimab	YuTuo^®^	WuXi Biologics/Guangzhou Gloria Pharmaceuticals Co Ltd; Arcus Biosciences, Inc; Gilead Sciences Inc; Taiho Pharmaceutical Co., Ltd	Human	IgG4 S228P	OmniAb^®^ Transgenic rat platform	Classical Hodgkin’s lymphoma
12	Serplulimab	Hetronifly^®^/HANSIZHUANG^®^	Shanghai Henlius Biotech, Inc.	Humanized	IgG4 S228P	mouse hybridoma	Microsatellite instability-high solid tumors
13	Pucotenlimab	Puyouheng^®^	Taizhou Hanzhong Biomedical Co., Ltd; HanX Biopharmaceuticals Inc; LEPU Biopharma/Akeso, Inc.	Humanized	IgG4 S228P/S254T/V308P/N434A	mouse hybridoma GPSmAb^®^	Metastatic microsatellite instability-high (MSI-H) or mismatch repair deficient (dMMR) advanced solid tumors
14	Enlonstobart	Enshuxing, 恩舒幸^®^	CSPC ZhongQi Pharmaceutical Technology Co. Ltd.	Human	IgG4 S228P	Phage display	Cervical cancer
15	Retifanlimab	Zynyz^®^	MacroGenics, Inc./Incyte Corporation/Zai Lab Limited	Humanized	IgG4 S228P	mouse hybridoma	Merkel cell carcinoma
16	Finotonlimab	Anyouping, 安佑平^®^	Sino Cell Technologies Inc.	Humanized	IgG4 S228P	Phage display antibody library	Head and neck cancer; Hepatocellular carcinoma

The Table includes INN name, commercial names, developers, V region engineering, isotype, origin of variable regions, and first approved therapeutic indication. The data was sourced from The Antibody Society (1) and the Immunogenetics database (IMGT) (15), and confirmed with the information published by the regulatory agencies that approved clinical use.

Although the 18 anti-PD-1 antibody-based drugs approved for human therapy have been developed using different engineering methods, have been characterized by diverse biochemical and cell-based assays, and have followed distinct regulatory pathways, all of them have successfully been validated in the clinic. Therefore, the review and discussion of the body of information published on these antibodies allowed us to draw some general trends that could serve as a reference for studying the mechanism of action (MOA) of anti-PD-1 therapeutic antibodies and/or designing new anti-PD-1-based drugs.

## 2 PD-1 signaling

PD-1 was first identified in the early 1990s, when it was found to be expressed during the induction of apoptosis in a T-cell hybridoma (20). However, it was later understood that the physiological role of PD-1 was closely related to immune regulation rather than to cell death (21). PD-1 is predominantly and highly expressed on activated T cells. However, it is also found on other immune cell populations, such as natural killer cells, B cells, and some populations of myeloid cells, although to a lower and more variable levels (22).

PD-1 is a co-inhibitory receptor that regulates the magnitude and duration of adaptive immune responses and is therefore a member of the immune checkpoint receptor family. PD-1 expression is induced in T cells only after T cell receptor (TCR) engagement (23), thereby acting as a negative feedback regulator that restrains T cell activation, proliferation and effector differentiation, thus limiting immunopathology and preserving peripheral tolerance (24). From an evolutionary angle, these inhibitory pathways probably arose to reconcile the need for effective pathogen control while preserving protection against tissue damage and autoimmunity. Nonetheless, under conditions of persistent exposure to antigens, such as those generated in chronic infection and cancer, sustained PD-1 signaling leads to T cell exhaustion, a state of T cell dysfunction characterized by reduced effector function, metabolic dysregulation and significant transcriptional and epigenetic reprogramming (25–27). The role of PD-1 in controlling immune homeostasis by mediating immune restraint in the context of chronic stimulation, provides the underlying rationale for therapeutic targeting of PD-1 and discussing the structural organization, ligand interactions and signaling mechanisms.

PD-1 signaling is initiated by interaction with its ligands PD-L1 and PD-L2, that have a dynamic expression pattern across hematopoietic and non-hematopoietic compartments. While PD-L1 is induced by a variety of inflammatory cues, including type I and type II interferons, and is expressed on antigen-presenting cells, stromal cells and a wide range of tumor cells, the expression pattern of PD-L2 is more limited and restricted primarily to professional antigen-presenting cells (28). Consequently, the distribution and density of PD-L1 and PD-L2 within the microenvironment of inflamed tissues and tumors are key determinants in the spatial and temporal activation of PD-1–dependent inhibitory signaling in T cells at sites of antigen recognition (29).

**Figure 2:**
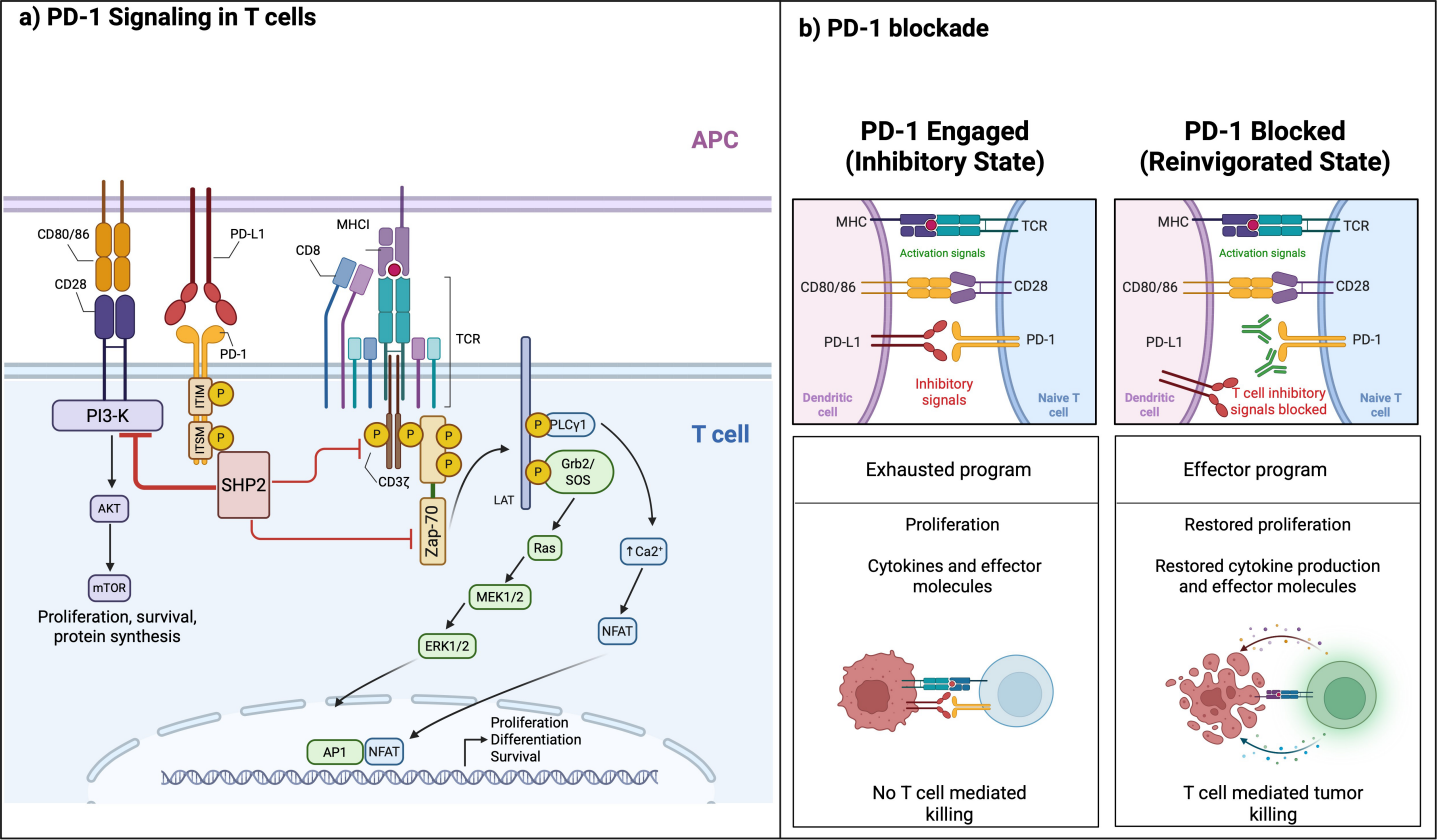
hPD-1 signaling and therapeutic reinvigoration. **(a)** PD-1 engagement. PD-1 interaction with PD-L1 recruits SHP2 to inhibitory microclusters, preferentially suppressing CD28–PI3K–AKT–mTOR and attenuating TCR–LAT–PLCγ1–RAS–ERK signaling. This uncouples NFAT from AP-1, enforces TOX/NR4A-dependent exhaustion, reduces mitochondrial fitness and glycolysis and diminishes proliferation, cytokine production and cytotoxicity, resulting in impaired tumor cell killing. **(b)** PD-1 blockade. There are two stages: Inhibitory state (left) when PD-1 on the T-cells binds PD-L1 in the effector cells, for instance, dendritic cells. Reinvigorated state (right) when the interaction between PD-1 and PD-L1 is blocked.

Upon ligand binding (**Figure 2**), the immunoreceptor tyrosine-based inhibitory motif (ITIM) and the immunoreceptor tyrosine-based switch motif (ITSM) of the intracellular domain of PD-1 are phosphorylated, leading to the preferential recruitment of the SHP2 phosphatase (30). At the immunological synapse, PD-1 forms micro-clusters that colocalize with costimulatory receptors, and nucleate SHP2-containing signalosomes, providing a biophysical basis for signal attenuation (30). SHP2 dephosphorylates the proximal signaling components at the immunological synapse, in which the co-stimulatory molecule CD28 has been identified as the most sensitive target. However, PD-1 signaling can also attenuate the phosphorylation of the TCR proximal signaling molecules including CD3ζ and ZAP70, therefore controlling TCR signaling (30–32). Inhibition of CD28 impairs PI3K recruitment and downstream AKT–mTOR activation, resulting in reduced glycolytic metabolism, diminished survival signaling and impaired clonal expansion.

Simultaneous attenuation of TCR-proximal signaling impairs LAT signalosome assembly, and thereby limits PLCγ1 activation, calcium flux and RAS–MEK–ERK pathway activation. Collectively, these effects reduce AP-1 availability and promote NFAT–AP-1 uncoupling, skewing T cells toward a dysfunctional transcriptional state. Due to these signaling alterations, PD-1 enforces a metabolic program characterized by reduced glycolysis, decreased mitochondrial fitness and increased reliance on fatty acid oxidation, thus limiting effector differentiation and cytokine production (33, 34). Sustained PD-1 signaling also promotes transcriptional and epigenetic changes that are associated with T cell exhaustion, including the induction of TOX and NR4A family members and stabilization of chromatin landscapes that limit functional reinvigoration (35, 36). The exhausted T cell state represents a distinct differentiation program characterized by progressive loss of effector function and the emergence of a TCF1^+^ progenitor subset that sustains responses to PD-1 blockade (30, 37).

These molecular and metabolic effects collectively reduce proliferation, cytokine production and cytotoxic activity and are only partially reversible following PD-1 blockade, a process that depends on CD28 expression on T cells and underlies both the clinical activity and the limitations of PD-1–targeted therapies (32). Therapeutic antibodies restore CD28 and TCR signaling, as well as effector function of this responsive T cell subset through the inhibition of ligand interactions with PD-1 and therefore the recruitment of SHP2 and all the downstream events (31, 32). However, the extent of T cell reinvigoration is limited by the availability of the ligands, antigen presentation processes and other suppressive mechanisms of the tumor microenvironment (29). These considerations provide a framework for understanding how antibody-mediated PD-1 blockade and meaningful therapeutic efficacy could be achieved.

## 3 PD-1 in immune escape

Tumors exploit the PD-1 pathway as one of their main ways to evade immune destruction and induce tolerance (38). Expression of PD-L1 in tumors occurs through two distinct mechanisms: adaptive immune-mediated and/or the oncogenic driven pathways. PD-L1 expression in many tumors is the result of adaptive immune resistance, in which interferon-γ secreted by CD8+ T cells that infiltrated to the tumor induce PD-L1 expression as a feedback mechanism to control excessive immune activation. Importantly, the expression of PD-L1 in tumor cells allows escape of immune destruction (29). In other cases, PD-L1 expression is driven by tumor-intrinsic oncogenic pathways, such as activation of MYC, ALK and PI3K–AKT signaling or loss of PTEN, independently of immune pressure (29, 39). Both the adaptive and constitutive programs are not mutually exclusive and more often overlap in regulating PD-L1 expression, resulting in generation of spatially heterogeneous inhibitory gradients based on PD-L1 expression that limit cytotoxic activity even in tumors with substantial infiltration of T cells.

Successful reinvigoration after PD-1 blockade requires functional antigen presentation and efficient interaction between antigen-presenting cells and T cells within the tumor microenvironment. The loss of expression of the major histocompatibility complex class I, which is often caused by mutations in β2-microglobulin or by defects in interferon-γ signaling, results in compromised antigen presentation (40). The presence of immunosuppressive myeloid populations in the tumor microenvironment, including tumor-associated macrophages and myeloid-derived suppressor cells, that express PD-L1, secrete inhibitory cytokines, and generate metabolically hostile and stressful environments by depleting nutrients and releasing reactive nitrogen and oxygen species, further inhibits T cell activation. Such mechanisms also impair dendritic cell maturation, thereby limiting co-stimulation and cross-presentation (29, 41). Together with impaired dendritic cell function and exclusion of T cells from tumor nests, limit productive T cell-tumor cell interactions and thus, reduce the efficacy of checkpoint inhibition (40).

The tumor microenvironment possesses other metabolic constraints, such as glucose depletion, hypoxia and accumulation of immunosuppressive metabolites, that further impact negatively in T cell fitness and limit reinvigoration following PD-1 blockade (42). Additionally, all the factors mentioned above act in combination with other inhibitory receptors, such as TIM-3, LAG-3 and TIGIT, which are often co-expressed on more terminally differentiated exhausted T cells and play a role in non-redundant circuits of suppression (43). Consistent with the notion of multiple mechanisms operating in immune escape, clinical responses to PD-1 blockade have been correlated with certain biomarkers such as the spatial distribution of PD-L1, interferon- γ response gene signature, preservation of MHC class I expression, T cell clonality and the presence of intratumor TCF1^+^ progenitor-like exhausted T cells (29). The mechanism of immune escape in different contexts emphasizes the importance of understanding the structural organization of PD-1 and its interaction with PD-L1 and PD-L2 as discussed below.

## 4 The PD-1 molecule and its interaction with PD-L1/L2

PD-1 is a 288-amino acid type I transmembrane glycoprotein composed of three domains, with a core molecular weight of 28–32 kDa that increases to 30–55 kDa due to glycosylation. The PD-1 extracellular domain (ECD) shares approximately 21-33% sequence identity with other members of the B7:CD28 family, such as CTLA-4, CD28, and ICOS (44). The transmembrane domain (TMD) is ~20 amino acids long with a propensity for dimerization that correlates with the ability of PD-1 to inhibit immune responses, antitumor immunity, cytotoxic T cell function, and autoimmune tissue destruction (45). The cytoplasmic domain (CTD) is approximately 95 amino acids containing the two conserved tyrosine-based signaling motifs, ITIM and ITSM, which are essential for downstream inhibitory signaling (see above) (20).

PD-L1 and PD-L2 are also transmembrane glycoproteins like other members of the B7 family. PD-L1 is approximately 40 kDa (290 amino acids) comprising three domains. The ECD folds into two Ig domains, a variable-like (IgV) and a constant-like (IgC) domain. The TMD is roughly 20 amino acids folded as a single helix that links the ECD with the CTD. The latter is ~30 amino acids and it is a critical mediator of cell-intrinsic signaling that protects cancer cells from immune-related stress (46). PD-L2 is shorter, having ~ 270 amino acids. The CTD is 30 amino acids in humans and has been reported to be involved in regulating B-1 cell functions, suppression of natural IgM production and secretion of IL-10 (47).

PD-L1 can further exist in soluble form (sPD-L1), produced mainly by myeloid-derived cells, such as monocytes, macrophages, and dendritic cells (DCs). sPD-L1 retains its ECD for interaction with PD-1 and can also suppress T-cell activation (48).

## 5 Structure of PD-1 ECD and molecular interactions with PD-L1/L2 ECDs

At the time of writing this review (March 2026), 37 PD-1 structures (**Table 2**) were available at the Protein Data Bank (PDB), including nine X-ray structures of hPD-1 and mouse PD-1 (mPD-1) ECDs free or in complex with PD-L1 or PD-L2 (49, 50, 52, 55, 63), one PD-1 structure solved by NMR (51), two structures of PD-1 mutants and 25 crystallographic X-ray coordinates of hPD-1 in complex with diverse antibodies. Analysis of this vast amount of structural information has provided a detailed picture of the architecture of hPD-1, differences with its mouse ortholog, have shed light on key molecular aspects of the interactions with PD-L1 and PD-L2, and has contributed to the understanding of the MOA mediated by anti-PD-1 therapeutic antibodies (52, 57, 76).

**Table 2:** PD-1 structures available in the protein data bank (PDB), including human and murine PD-1, PD-1 complexes with PD-L1 or PD-L2, and PD-1 bound to antibodies.

#	PDB ID	Structure	Resolution (Å)	Method	Release date	References
1	1NPU	mPD-1 ECD	2.00	X-ray	2004/03/23	(49)
2	3BIK	mPD-1/hPD-L1 complex	2.65	X-ray	2008/02/26	(50)
3	3SBW	mPD-1 mutant and hPD-L1 complex	2.28	X-ray	2011/07/13	TBP
4	3RRQ	hPD-1 ECD	2.10	X-ray	2012/05/16	TBP
5	2M2D	hPD-1 ECD	-–	NMR	2013/02/27	(51)
6	4ZQK	hPD-1/hPD-L1 complex	2.45	X-ray	2015/11/04	(52)
7	5C3T	hPD-L1/PD-1-binding domain	1.80	X-ray	2015/11/04	(52)
8	5DK3	Pembrolizumab (IgG4 mAb)	2.28	X-ray	2015/11/18	(53)
9	5JXE	hPD-1/pembrolizumab Fab complex	2.90	X-ray	2016/08/10	(54)
10	5IUS	hPD-L1/PD-1 mutant complex	2.89	X-ray	2016/09/28	(55)
11	5B8C	hPD-1/pembrolizumab Fv complex	2.15	X-ray	2016/10/26	(56)
12	5GGR	hPD-1/nivolumab Fab complex	3.30	X-ray	2016/11/09	(57)
13	5GGS	hPD-1/pembrolizumab Fab complex	2.00	X-ray	2016/11/09	(57)
14	5WT9	hPD-1/nivolumab Fab complex	2.40	X-ray	2017/02/15	(58)
15	6HIG	hPD-1/NBO1a Fab complex	2.20	X-ray	2019/06/05	(59)
16	6JBT	hPD-1/toripalimab Fab complex	2.47	X-ray	2019/06/19	(60)
17	6JJP	hPD-1/MW11-h317 (Penpulimab) Fab complex	2.90	X-ray	2019/10/30	(61)
18	6J14	hPD-1/GY-14 Fab complex	1.40	X-ray	2019/11/06	(62)
19	6J15	hPD-1/GY-5 Fab complex	2.60	X-ray	2019/11/06	(62)
20	6UMT	hPD-1/hPD-L2 complex	1.99	X-ray	2019/11/27	(63)
21	6UMU	hPD-1 triple mutant	1.18	X-ray	2019/11/27	(63)
22	6UMV	hPD-1 double mutant	1.42	X-ray	2019/11/27	(63)
23	6K0Y	hPD-1/mAb059c Fab complex	1.70	X-ray	2019/12/11	(64)
24	7BXA	hPD-1/tislelizumab Fab complex	3.32	X-ray	2020/06/10	(65)
25	6XKR	hPD-1/sasanlimab Fab complex	2.59	X-ray	2020/09/09	(66)
26	7CU5	hPD-1/camrelizumab complex	2.81	X-ray	2020/10/14	(67)
27	7CGW	hPD-1/tislelizumab Fab complex	3.20	X-ray	2021/04/07	(68)
28	7E9B	hPD-1/HLX10 Fab complex	1.78	X-ray	2021/07/28	(69)
29	7VUX	hPD-1/609A Fab complex	1.64	X-ray	2021/11/17	(70)
30	7WVM	hPD-1/cemiplimab complex	3.40	X-ray	2022/04/20	(71)
31	7WSL	hPD-1/dostarlimab complex	1.53	X-ray	2022/08/24	(72)
32	8AS0	hPD-1/D12 Fab complex	3.50	X-ray	2022/11/23	(73)
33	8GY5	hPD-1/cemiplimab Fab complex	1.98	X-ray	2023/04/10	TBP
34	8EQ6	hPD-1/clone 19 Fab complex	1.65	X-ray	2023/10/11	(74)
35	9EHT	hPD-1/retifanlimab Fab complex	1.54	X-ray	2025/03/12	TBP
36	8U31	hPD-1/h1340.CC Fab complex	2.73	X-ray	2024/06/19	(75)
37	8U32	hPD-1/h1340.SA.LV Fab complex	2.51	X-ray	2024/06/19	(75)

For each entry, the PDB identifier, structure description, resolution, experimental method, release date, and reference are indicated. TBP (To be published).

**Figure 3:**
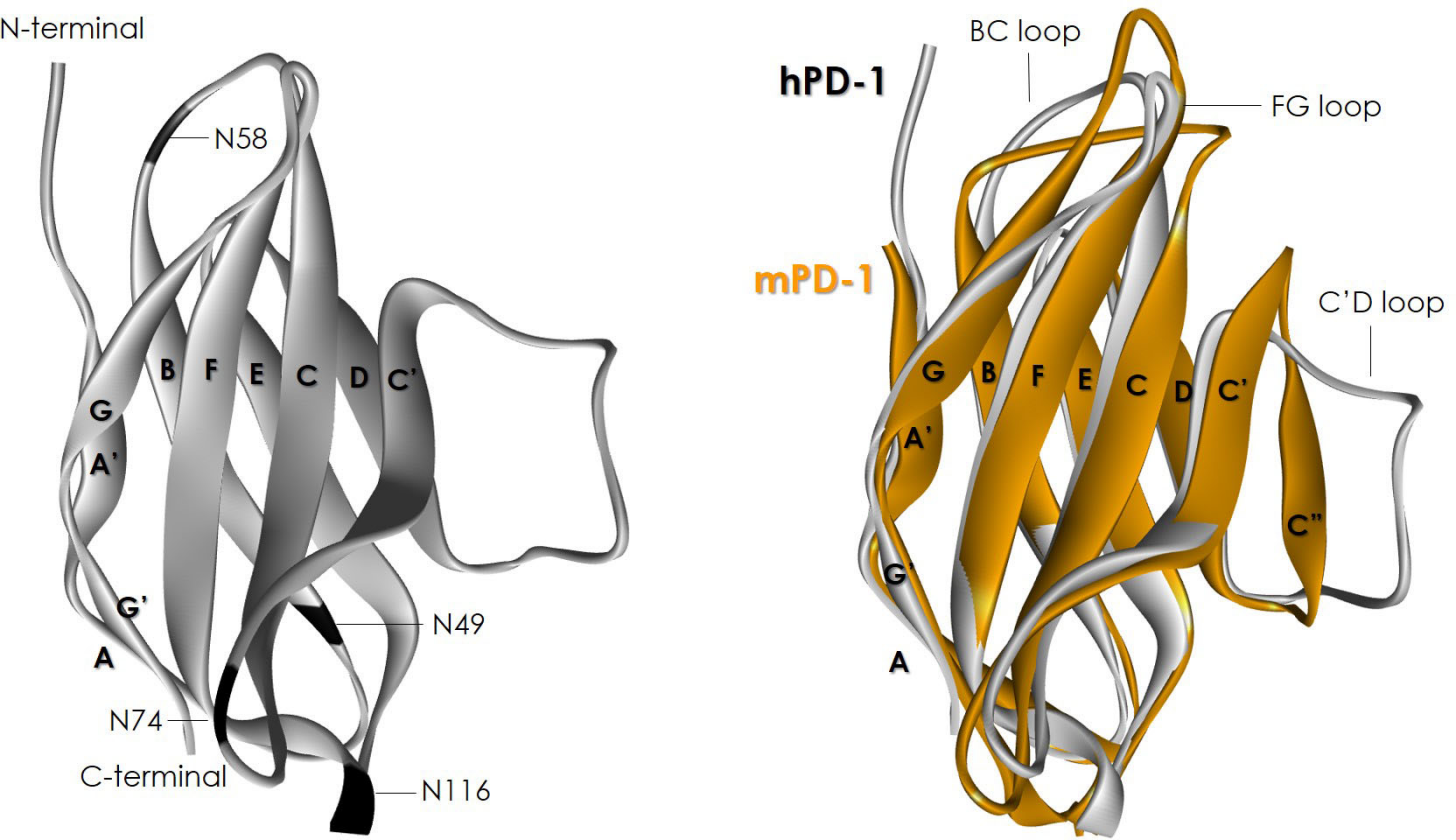
Ribbon representation of hPD-1 ECD (left) and superposition with mPD-1 (right). Both panels show the front β-sheet formed by β-strands CC′FGG’ plus the FG loop binds PD-L1. The back β-sheet is formed by β-strands AA’BED and is not involved in the interaction with PD-L1. The four N-glycosylation sites, N49, N58, N74, and N116, are indicated in black in hPD-1. The hPD-1 figure was generated with the coordinates from PDB ID: 5GGS. The mPD-1 was generated with the PDB ID: 1NPU. Note the difference in conformation at the BC, FG and C’D loop conformations between hPD-1 and mPD-1.

PD-1 ECD folds with an immunoglobulin-variable (IgV) topology (**Figure 3**) built by 10 β-strands: A’, A, B, C, C’, D, E, F, G and G’, with a disulfide bridge between Cys54 and Cys123. The front β-sheet binds PD-L1/L2 and is formed by β-strands CC′FGG’. The back β-sheet is formed by β-strands AA’BED. Four N-glycosylation sites, namely: N49, N58, N74, and N116, decorate the ECD. All of them are occupied with glycans, as mutation of each of these sites leads to a significant reduction in the molecular weight according to SDS-PAGE (77). The N-glycosylation at residue N58 has been reported to be important for PD-1 binding to PD-L1 and is involved in the interaction with some approved anti-PD-1 antibodies. On the other hand, the NMR structure of PD-1 (51) and comparison of the PD-1 X-ray structures free and bound to diverse antibodies have indicated that the N-terminal, C’D, BC and FG loops at the membrane-distal region of PD-1 are highly flexible and play an important role in the interaction with the antibodies, as discussed below.

The hPD-1 and mPD-1 ECDs are relatively conserved (65% homology), with the region of the C’D loop being one of the least conserved (51). The C’D loop of hPD-1 is relatively long and flexible, whereas mPD-1 adds the β-strand C” to link β-strands C’ and D. Further, a proline residue in hPD-1 twists the FG loop and changes the conformation of the BC loop with respect to mPD-1, also affecting the conformation of the DE loop. These structural differences are responsible for the remarkable differences in the affinity of hPD-1 and mPD-1 for their ligands and help explain why most of the antibodies targeting hPD-1 do not cross-react with the mouse ortholog.

**Figure 4:**
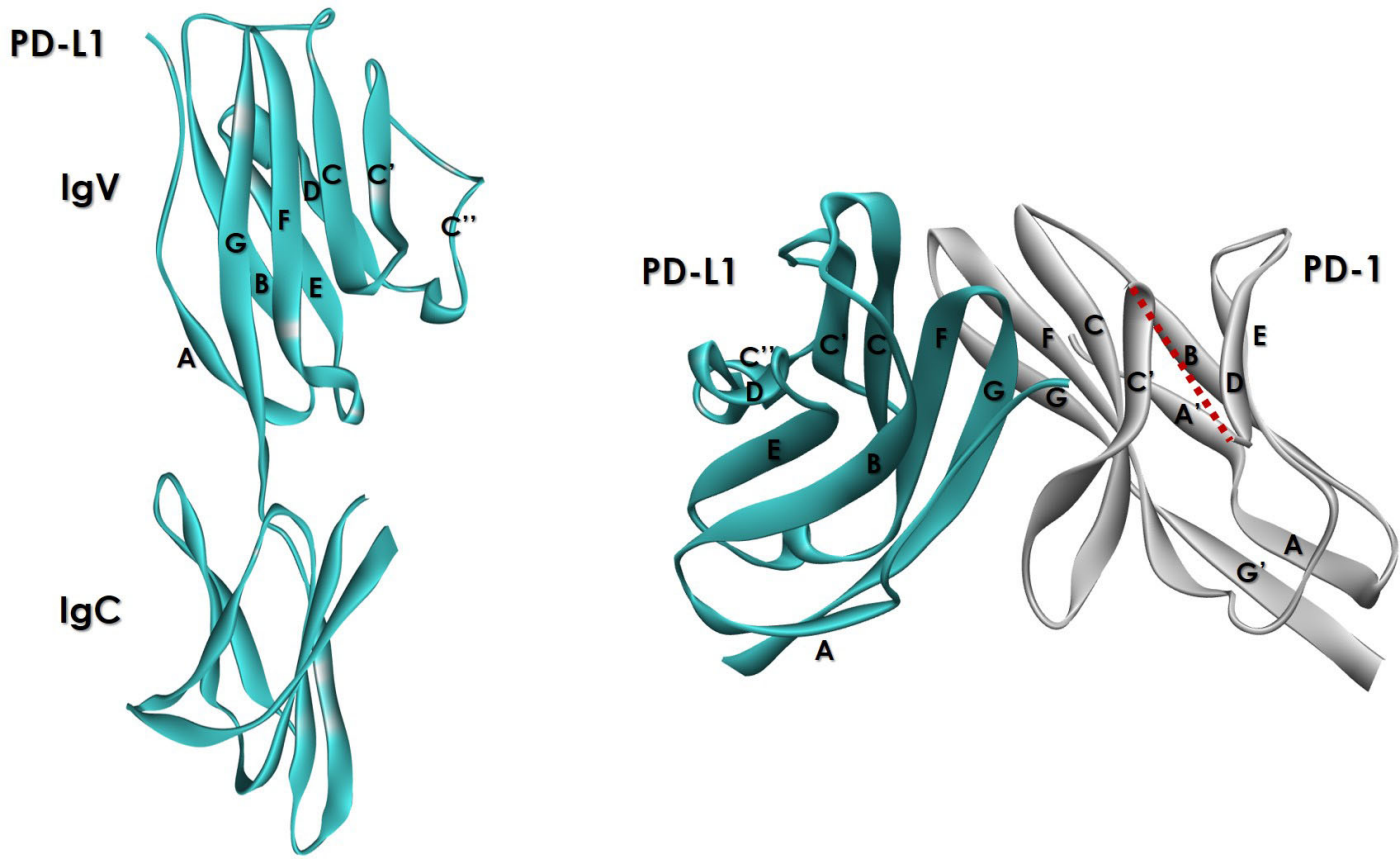
Ribbon representation of hPD-L1 ECD (left). The hPD-1 binding domain (left panel) is folded with an IgV topology (PDB ID: 3BIK). The front β-sheet is formed by β-strands AGFCC′C″. The back β-sheet is formed by β-strands BED. The right panel (PDB ID: 4ZQK) shows the complex formed by hPD-L1 (blue) and hPD-1 ECDs. The C’D loop is not visible in this structure and is represented with a discontinuous red line.

The PD-L1 ECD IgV domain is responsible for the interaction with PD-1. Like PD-1 ECD, the PD-L1 ECD IgV domain consists of two antiparallel β-sheets (**Figure 4**) formed by nine β-strands and a disulfide bond between Cys40 and Cys114 (52). The front β-sheet of PD-L1, formed by β-strands AGFCC′C″, binds the front β-sheet of PD-1. The back β-sheet is structured by β-strands BED and does not participate in the interaction with PD-1. The PD-1:PD-L1 dimer assembles upon binding in an orthogonal orientation resembling the quaternary structure of an antibody variable fragment (Fv).

The sequences and structures of PD-L1 and PD-L2 are similar, with the main difference lying in the presence of an alanine versus tryptophan residue in PD-L1 position 121 and in PD-L2 position 110 (78, 79). The tryptophan side chain in PD-L1 is buried in the hydrophobic core of the protein and does not contribute to the interaction with PD-1. The corresponding tryptophan side chain in PD-L2 is exposed and oriented toward the PD-1 core in the PD-1/PD-L2 complex. Together with Tyr110, it opens an even larger and deeper cavity within PD-1 compared to that induced by PD-L1 binding, resulting in a more stable binding between these two proteins and thus, tighter binding of the PD-1 with PD-L2 than that of PD-L1. The structural features and binding affinity of the PD-1:PD-L1/L2 interaction have provided the foundations for understanding how therapeutic antibodies engage PD-1 and how different mechanisms of binding could influence their functional properties, reviewed in the following sections.

## 6 V region engineering of the anti-PD-1 antibodies

The specificity and affinity of the antibodies for PD-1 reside in their V regions, one from the heavy chain (V_H_) and another from the light chain (V_L_). Ten of the 16 antibodies (63%) compiled in **Table 1** have humanized V regions. The remaining six are fully human antibodies obtained from transgenic mice or rats, or are selected via phage or mammalian display technologies. All the antibodies except prolgolimab and zimberelimab have kappa light chains. The former bears a hybrid light chain composed of a lambda variable and a constant kappa domain. Zimberelimab has a full lambda light chain.

**Figure 5:**
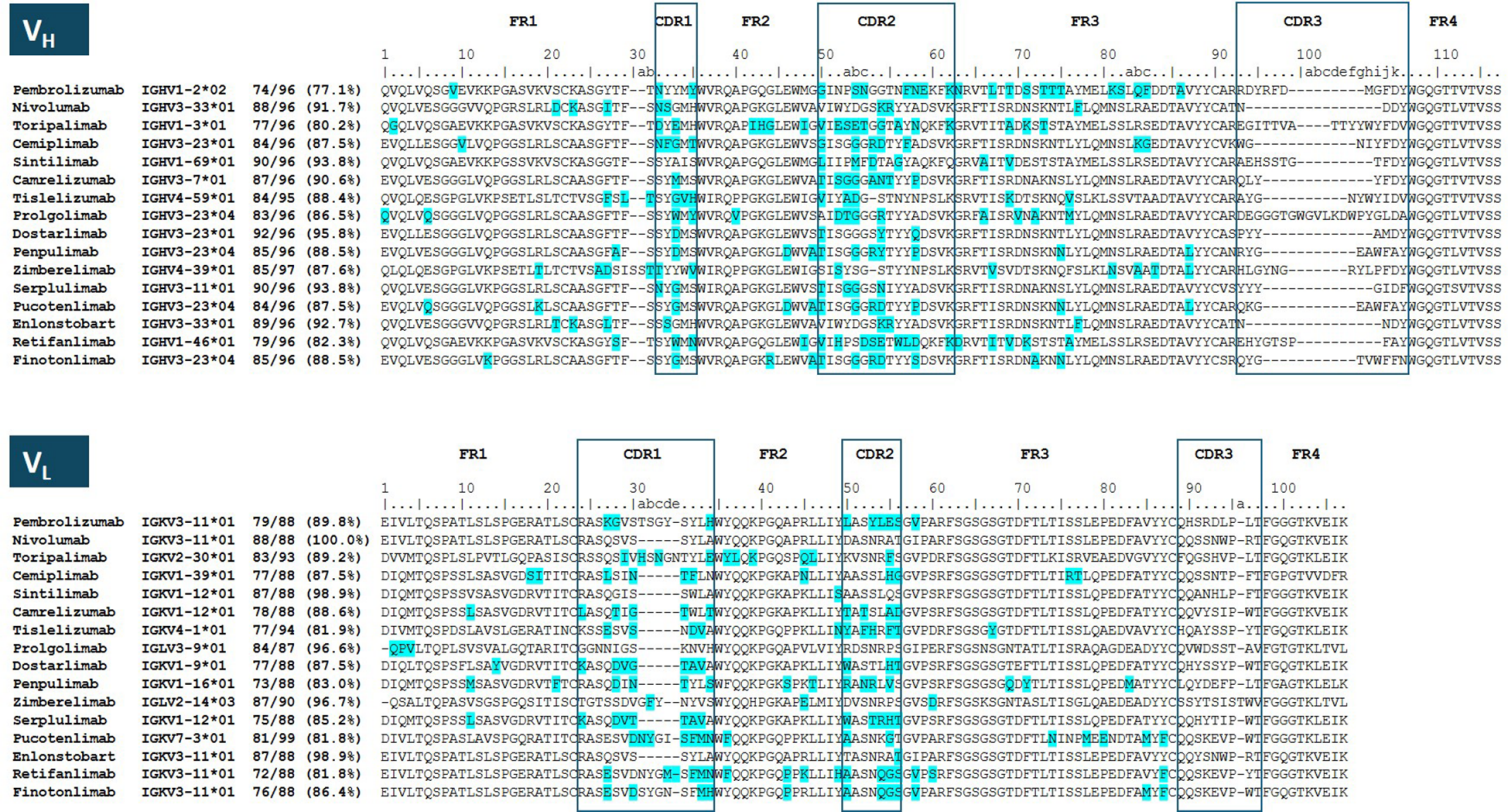
Multiple sequence alignment of V_H_ and VL of clinically approved anti–PD-1 antibodies. Numbering according to Kabat (80). Antibody sequences were retrieved from the IGMT information system^®^ (15), and DrugBank (81). The name of the IGV germline genes corresponds to IMGT reference nomenclature. Framework regions (FR1–FR4) and complementarity-determining regions (CDR1–CDR3) are indicated with boxes in the alignments. Amino acids in the antibody sequence differing from the corresponding IGV germline gene are highlighted in blue. Percent identity values were calculated across the V-gene–encoded region spanning FR1 to FR3 (including CDR1 and CDR2), while CDR3 and FR4 were excluded from the analysis.

The sequence alignments of V_H_ and V_L_ of the antibodies listed in **Table 1** are shown in **Figure 5**. The percentage of identity with respect to the human IGV germline genes, defined as human content (82, 83), ranges in V_H_ from 77% in pembrolizumab to 96% in dostarlimab. The V_L_ region has a higher human content, ranging from 82% in retifanlimab and tislelizumab to 100% in nivolumab. Three out of seven human IGHV germline gene families (IGHV1, IGHV3 and IGHV4) were utilized either as human Framework (FR) acceptors for Complementarity Determining Regions (CDRs) grafting of the non-human parental antibodies during humanization or were selected directly from the human antibody repertoire in the case of fully human antibodies. FRs derived from the IGHV2, IGHV5, IGHV6 and IGHV7 gene families are not represented among the currently approved anti-PD-1 antibodies.

The CDR-3 of V_H_ (HCDR3) plays a central role in determining antibody specificity due to its placement at the center of the antigen-binding site and the diversity generated through V(D)J recombination and junctional diversification (84). These diversification mechanisms generate human HCDR3 loops with a Gaussian length distribution ranging from 2 to 26 amino acids with an average length of 12 amino acids (85, 86). The HCDR3 of the anti-PD-1 antibodies range from 3 to 20 amino acids (Kabat numbering) (87), with an average length of seven amino acids. Thus, the HCDR3 of anti-PD-1 antibodies tends to be shorter than the average HCDR-3 length in humans, with the majority of HCDR3 loops falling within a compact range of 4–8 amino acids. Two antibodies have long HCDR3 loops, toripalimab (16 amino acids) and prolgolimab (20 amino acids). The long HCDR3 of toripalimab plays an important role in the interaction with PD-1 (see below). No structure for prolgolimab is currently available and it could not currently be elucidated how the long HCDR3 and the hybrid lambda/kappa light chain of prolgolimab participate in the interaction with PD-1.

The lengths of the CDR-2 and 3 of V_L_ (LCDR2 and LCDR3) are conserved in all the sequences except zimberelimab, which has an insertion in the CDR-3 of V_L_ (LCDR3). The LCDR1 length is highly variable, covering six lengths, with toripalimab having the longest LCDR1. Overall, V_L_ is more diverse than V_H_ in terms of the germline gene family usage. Five (IGKV1, IGKV2, IGKV3, IGKV4, and IGKV7) out of seven germline IGKV gene families encode the anti-PD-1 antibodies, with 12 out of 16 (75%) antibodies having IGKV1 and IGKV3 family genes. Several antibodies are encoded by the IGKV1–39 and IGKV1-12, as well as IGKV3–11 and IGKV3–20 germline genes, which are among the most frequently utilized in the human antibody repertoire (88) and have been the substrate for building phage-displayed human libraries for antibody discovery (89). The recurrent selection of these IGV germline genes across independently developed anti-PD-1 antibodies is consistent with the favorable developability profile of the amino acid sequence they encode (90, 91).

## 7 Isotype of the anti-PD-1 antibodies

In addition to the V regions impact on the developability profile of antibodies, the choice of the Fc also defines their physiochemical and biological properties. Human IgG has evolved four isotypes: IgG1, IgG2, IgG3, and IgG4 (92) with different Fc sequences and differential capacity to elicit effector functions such as ADCC, CDC and ADCP. IgG1 is the isotype eliciting stronger effector functions based on the selective Fcγ receptors (FcγR) interactions on distinct immune cell populations, as well as the ability to bind C1q, an initial protein in the complement pathway leading to a ‘‘cascade’’ of events that results in the formation of the MAC and the induction of tumor cell killing. In contrast, IgG4 elicits minimal effector functions, and since the MOA of anti-PD-1 antibodies is by blocking the interaction with PD-L1/L2 with minimal Fc-mediated effector functions, it has been the isotype of choice to engineer the vast majority (14 out of 16; 87%) of the anti-PD-1 antibodies (see **Table 1**).

Yet, IgG4 tends to rearrange *in vivo* and exchange antigen-binding fragments (Fab) in which one heavy chain and one light chain of one antibody can associate with other half molecules (93). To mitigate this liability of the IgG4 isotype, a mutation of serine to proline in position 228 (S228P) of the hinge region has been introduced in all the 14 antibodies having the IgG4 isotype. This mutation abolishes the chain swap and hence, the heterogeneity of human IgG4 antibodies (93, 94).

Two IgG4 antibodies listed in **Table 1**, tislelizumab and pucotenlimab, have additional mutations in the Fc region. Pucotenlimab has three mutations: S254T/V308P/N434A, in addition to S228P. These mutations increase binding to both human and nonhuman primate (NHP) Neonatal Fc receptor (FcRn) at the acidic pH of the endosome, with the consequent increase in the half-life of the antibody (95). Therefore, these mutations allow the simultaneous modulation of serum half-life, tissue distribution and attenuated effector functions. Tislelizumab, on the other hand, has the mutations E233P/F234V/L235A/D265A, besides S228P, which stabilize the hinge region decreasing the binding to FcγRs (68, 96).

Although IgG4 has been the isotype of choice for engineering most of the anti-PD-1 antibodies, prolgolimab and penpulimab, have IgG1 isotypes. The human IgG1 isotype has some advantages with respect to IgG4. IgG1 is more stable than IgG4 with a thermal stability of 64°C (IgG4) *vs* 68°C (IgG1) of the C_H_2 domain – the least stable domain of the IgG (97). Further, IgG1 generally exhibits higher solubility than IgG4 in common pharmaceutical buffers and has lower viscosity and self-interaction than IgG4, which can facilitate the formulation of the molecule at high concentrations. Also, IgG1 has a longer hinge region than IgG4 (15 amino acids *vs.* 12 amino acids, respectively). A long hinge region confers greater flexibility to the antibody, likely facilitating binding of diverse target configurations on the cell surface. However, IgG1 induces strong ADCC, CDC, and ADCP responses which is a drawback when blocking with minimal effector functions is the desired MOA (98).

Thus, the two antibodies engineered with an IgG1 isotype carry the mutations L234A/L235A, known as “LALA” variant (99). The LALA mutations in the C_H_2 domain of the IgG1 have been described as one of the most potent and frequently used modifications to reduce binding to the FcγRs as well as to C1q (100), leading to attenuated effector functions (98, 101, 102). Comparative preclinical studies of prolgolimab effector functions with IgG4 antibodies such as nivolumab and pembrolizumab have shown that none of the antibodies had undesirable ADCC. In these studies, prolgolimab showed higher PD-1 receptor occupancy and T-cell activation, with higher tumor growth inhibition in mouse models than pembrolizumab, together with lower propensity to activate ADCP (103). Penpulimab carries an additional mutation, G237A. This mutation abrogates the effector functions further, without affecting the half-life of this antibody, which is around 22 days, similar to that of pembrolizumab (104, 105).

## 8 Immunogenicity

Immunogenicity is an important concern during the preclinical and clinical development of biotherapeutics as it may render the drug inactive or lead to unwanted adverse effects. It has been documented (106) that humanized antibodies generally tend to elicit higher immunogenic responses than fully human antibodies. Since most of the antibodies listed in **Table 1** are humanized molecules, we sought to compile and examine the reported incidence of anti-drug antibodies (ADAs) in humanized versus fully human anti-PD-1 antibodies.

The incidence of non-neutralizing ADAs varies significantly across the antibodies (**Table 3**), ranging from no observed ADAs in zimberelimab and prolgolimab to 20.4% in camrelizumab. Fully human antibodies - except nivolumab with 11% - elicited ADAs below 2.5%. Seven out of nine (~80%) of the humanized antibodies elicited ADAs above 2.5%, with only penpulimab and retifanlimab having ADA incidence of 1.8%. Thus, the reported data suggests a trend toward higher ADA incidence in humanized compared to fully human anti-PD-1 antibodies. However, this observation should be interpreted with caution, as ADA incidence rates are not directly comparable across studies due to differences in the assay design, validation strategies, sample handling, and study populations, all of which can significantly influence ADA detection (122, 123).

**Table 3:** Incidence of anti-drug antibodies (ADAs) and neutralizing antibodies (NAbs) for approved therapeutic anti-PD-1 antibodies based on data from representative clinical trials.

Antibody	Clinical study	ClinicalTrials.gov ID	Dosage schedule	% ADAs	% NAbs	Reference
Pembrolizumab	Study of Pembrolizumab (MK-3475) Versus Investigator’s Choice Standard Therapy for Participants with Advanced Esophageal/​ Esophagogastric Junction Carcinoma That Progressed After First-Line Therapy (MK-3475-181/​KEYNOTE-181)	NCT02564263	200 mg Q3W	3.9% (11/283)	0%	(107)
Nivolumab	A Study of MDX-1106 in Patients with Selected Refractory or Relapsed Malignancies (MDX1106-01)]/CHECKMATE-037/CHECKMATE-017/CHECKMATE-057/CHECKMATE-066/CHECKMATE-025/CHECKMATE-067.	NCT00441337 NCT01721746 NCT01642004 NCT01673867 NCT01721772 NCT01668784 NCT01844505	240 mg Q2W/480 mg Q4W/3 mg/kg Q2W	11% (229/2085)	0.7% (15/2085)	(108)
Toripalimab	JUPITER-02/POLARIS-02	NCT03581786 NCT02915432	240 mg Q3W (combo)/ 3 mg/kg Q2W (mono)	3.4% (5/146) in combination therapy	Not reported	(109)
Cemiplimab	R2810-ONC-1423/R2810-ONC-1540	NCT02383212 NCT02760498	350 mg Q3W or 3 mg/kg Q2W	1.9-2.5%	0%	(110)
Camrelizumab	Evaluate the Safety and Tolerability of Using the SHR-1210 by Advanced Solid Tumor Subjects	NCT02721589	1mg/kg, 3mg/kg, 10mg/kg/200mg Q2W	20.4% (10/49)	Not reported	(111)
Tislelizumab	Consolidated data from 17 studies.	NCT02407990 NCT03209973 NCT03430843	200 mg Q3W/2–5 mg/kg Q2W/Q3W	18.7% (84/451)- 19.2% (731/3815)	0.3% (1/451)	(112, 113)
Prolgolimab	Prolgolimab 250 mg Q3W in Patients with Unresectable or Metastatic Melanoma (FLAT)/International Trial of the Efficacy and Safety of BCD-100 in Patients with Melanoma (MIRACULUM)	NCT05783882 NCT03269565	250 mg Q3W (dosis fija)/1 mg/kg Q2W	0% (0/114)	Not reported	(114)
Dostarlimab	Study of TSR-042, an Anti-programmed Cell Death-1 Receptor (PD-1) Monoclonal Antibody, in Participants with Advanced Solid Tumors (GARNET)	NCT02715284	500 mg Q3W for 4 doses → 1000 mg Q6W	2.5% (8/315) - RTD	1.3% (4/315)	(115)
Penpulimab	Evaluate the Efficacy and Safety of Anti-PD-1 Antibody AK105 in Patients with Selected Advanced Solid Tumors	NCT04172506	200 mg Q2W	1.8% (1/55)	1.8% (1/55, NAb+)	(116)
Zimberelimab	Study of GLS-010 Injection in the Treatment of Classical Hodgkin’s Lymphoma	NCT03655483	240 mg Q2W	No immunogenicity was observed	Not reported	(117)
Serplulimab	Phase 1 Study of HLX10, in Patients with Advanced Solid Tumors/ A Clinical Study to Evaluate HLX10 Monotherapy for the Treatment of MSI-H or dMMR Solid Tumors That Failed to Respond to Standard Therapy	NCT03468751 NCT03941574	200 mg Q2W, 300 mg Q3W, 400 mg Q4W, 600 mg Q6W/3 mg/kg Q2W	4.6% (5/108) en MSI-H; 18.9% (7/37) en fixed doses	0% (0/108)	(118)
Pucotenlimab	A Phase Ib Study of HX008 in Patients with Advanced Solid Tumors	NCT04825392 CTR20180125 (Chinese Clinical Trial Registry)	1, 3, 10 mg/kg Q3W/ 200 mg Q3W	3.3% (1/30)	Not reported	(119)
Enlonstobart	SG001 Injection for Patients with Relapsed or Metastatic Uterine Cervical Cancer	NCT04886700	240 mg Q2W	1.0% (1/97)	Not reported	(120)
Retifanlimab	A Study of INCMGA00012 in Participants with Selected Solid Tumors (POD1UM-203)	NCT03679767	500 mg Q4W	1.8% (2/113)	Not reported	(121)

Study identifiers (ClinicalTrials.gov or Chinese Clinical Trial Registry), dosing regimens, and corresponding references are indicated. ADA incidence rates should be interpreted with caution, as they are not directly comparable across studies due to differences in assay methodologies, study design, and patient populations.

On the other hand, none of the antibodies show high levels of neutralizing ADAs, which can render the antibody inactive. For instance, nivolumab elicited 11% of non-neutralizing ADAs in monotherapy, and when used in combination with ipilimumab the level of detected ADAs increases to 43% (124, 125), despite the fact that nivolumab has a high human content in V_H_ with only five mutations with respect to the germline gene, a very short HCDR3 (4 amino acids), and the highest human content in V_L_ with 100% identity with respect to the human germline gene configuration (see **Figure 5**). However, nivolumab has shown 0.7% of neutralizing ADAs in contrast with the humanized antibodies dostarlimab and penpulimab, which elicited 1.3% and 1.8% of neutralizing ADAs, respectively.

Having discussed the V and Fc engineering of the approved anti-PD-1 antibodies, and the impact on immunogenicity, in the next sections we review their functional features including affinity for hPD-1, hPD-1:PD-L1 blocking activity, and mechanism of hPD-1 binding.

## 9 Affinity of anti-PD-1 antibodies

A study (126) of the relationship between affinity and efficacy proposed that anti-PD-1 efficacy follows an expected receptor occupancy model, where the treatment can be tuned by the dose-affinity relationship. In this work (126), anti-PD-1 antibodies with an affinity range from 0.02 to 15 nM were tested in a single lineage of a syngeneic, immunocompetent mouse model, revealing that there is a threshold affinity required for maximum efficacy at a given dose. Below the affinity threshold of around 20-fold the affinity of hPD-1 for PD-L1/L2, all antibodies showed similar maximal efficacy and an increase in the dose compensated for a relatively weak affinity. Remarkably, beyond the affinity threshold, an increase in affinity may not translate into improved efficacy (126).

The affinity of the interaction of hPD-1 with hPD-L1/L2, reported as dissociation constant (K_D_), varies depending on the method used to determine it. Measurements by biolayer interferometry (BLI) have yielded K_D_ values of the hPD-1:PD-L1 and hPD-1:PD-L2 binding of 4, 100 ± 110 nM and 500 ± 82 nM, respectively (63). By surface plasmon resonance (SPR), the reported values are 8, 200 ± 100 nM for hPD-1/PD-L1 and 2, 300 ± 100 nM for PD-1/PD-L2 (51), whereas by isothermal titration calorimetry (ITC) the K_D_ values are 2, 200 nM and 450 nM, respectively (51). Although differences exist in the quantitative K_D_ values depending on the measurement method, the affinity of the interaction between hPD-1 and its ligands falls in the high nM to low µM range, with hPD-1:PD-L2 being 4-8-fold tighter than hPD-1:PD-L1.

The affinity of the interaction of hPD-1 for antibodies has also been assessed by diverse methods including ELISA, ITC, BLI, SPR, and KynExA (127). Due to the variability across assay design, instrumentation and experimental conditions, the reported affinity data for the same antibody varies among reports and should be interpreted carefully, focusing on general trends rather than direct quantitative comparisons among diverse platforms.

In **Table 4**, column 2, we compiled the K_D_ values as determined by SPR, which is the gold standard in field. It should be noted thought, that even within a methodology such as SPR, K_D_ measurements for the same antibody vary (138) as they depend on the density of ligand attached to the sensor chip (138), the capture method such as anti-IgG or Protein A (139) or whether the antibody is captured on the sensor chip or flowed over the captured ligand on the sensor chip. To avoid biases due to differences in SPR protocols, column 3 of **Table 4** compiles K_D_ values measured by Brown et al. (127) for 8 out of the 16 clinically approved antibodies. Brown et al. compared K_D_ measurements using diverse platforms and protocols. We focused on the Biacore 8K instrument, equipped with a CM5 sensor chip coated with a goat anti-human IgG Fc-specific polyclonal antibody as the anti-PD-1 antibody capture reagent, and hPD-1 His-tagged monomer injected over the flow cells.

**Table 4:** Reported K_D_ values determined by surface plasmon resonance (SPR) and biolayer interferometry (BLI), together with reported IC_50_ values from hPD-1:PD-L1 blocking assays.

Antibody	SPR	Brown et al. (127)	BLI	hPD-1: PD-L1 blockage
	KD (nM)	KD (nM)	KD (nM)	IC_50_ (nM)
Pembrolizumab	0.72 (104)*, 1.25 (128) 4.07 (68)**, 9.60 (129)	5.30	0.029 (130), 8.04 (69)	0.63 (13)
Nivolumab	0.54 (104)*, 1.16 (131), 3.05 (69), 6.10 (129), 6.92 (68)**	7.40	11.90 (69)	6.60 (69)
Toripalimab	0.10 (128)	–	3.85 (69)	1.30 (128)
Cemiplimab	1.68 (71)	1.90	–	0.60 (71)
Sintilimab	0.25 (132), 0.32 (69)	<0.21	2.09 (69)	29.2 (132)
Camrelizumab	3.00 (133), 4.80 (71)	4.50	–	0.70 (71)
Tislelizumab	0.11 (68)**	0.26	–	–
Prolgolimab	–	–	0.19 (134)	–
Dostarlimab	0.30 (135)	1.70	–	1.80 (135)
Penpulimab	0.59 (104)*	–	–	–
Zimberelimab	0.18 (131)	–	–	0.58 (131)
Serplulimab	0.32 (69)	–	2.42 (69)	4.36 (69)
Pucotenlimab	0.08 (69)	–	–	0.49 (69)
Enlonstobart	0.22 (136)	–	–	9.80 (136)
Retifanlimab	0.60 (129)	0.65	–	–
Finotonlimab	–	–	–	0.95 (137)

* hPD-1 ECD fused to human IgG Fc domain (PD-1-hFc) immobilized on a CM5 sensor chip. The full IgG was flowed through the sensor chip. Hence it measured avidity rather than true affinity.

** hPD-1 was immobilized on the sensor chip but the ligand was the Fab, flowed through the sensor chip. Hence, it does not measure the cooperative binding effect of the bivalent antibody, instead estimate true affinity.

The K_D_ values reported for pembrolizumab have been somewhat controversial, with 0.029 nM initially estimated by BLI (130). More recent reports on SPR-based measurements compiled in **Table 4**, indicate that the K_D_ values of pembrolizumab are between 0.72 nM (104) and 9.60 nM (129). The report of 0.72 nM used hPD-1 as capture reagent while pembrolizumab was flowed over the sensor chip, thus this K_D_ value seems to measure the cooperativity effect of binding (avidity) rather than affinity. The affinity measurements of nivolumab cover a range of 0.54 nM (104) to 6.92 nM (68). Like in pembrolizumab, the K_D_ value of 0.54 nM for nivolumab measured avidity rather than affinity, as hPD-1 was the capture reagent and nivolumab was flowed over the sensor chip. Therefore, if only K_D_ measurements using SPR, capturing the antibodies on the sensor chip and hPD-1 flowed through it are considered, the affinity of both pembrolizumab and nivolumab are in a single-digit nM, which is consistent with the K_D_ reported by Brown et al. (127) of 5.30 and 7.40 nM for pembrolizumab and nivolumab, respectively.

**Table 4**, column 4, further lists the K_D_ values reported for five antibodies determined by the Issafrads et al. (69) using BLI. The K_D_ values for toripalimab and sintilimab differ significatively depending on the method. Using SPR, the K_D_ values reported in the literature are 0.10 and 0.25 nM for toripalimab and sintilimab, respectively (128, 132). Using BLI, the reported K_D_ values are 3.85 and 2.09 nM, respectively. Issafrads et al. (69) also reported SPR measurement for sintilimab and nivolumab of 0.32 and 3.05 nM, respectively. On the other hand, Brown et al. (127) only reported the K_D_ for sintilimab, which is <0.21 nM. Hence, we can safely assume that the BLI measurements underestimated the K_D_ values and that both toripalimab and sintilimab have sub-nM affinity.

Cemiplimab and camrelizumab have consistently K_D_ values in the low nM range by different authors – including Brown et al. (127). All the other antibodies have sub-nM affinities. Therefore, as a general trend and despite quantitative differences among the K_D_ values reported in the literature by different laboratories for the same antibody, the affinity of anti-PD-1 antibodies compiled in **Table 4** falls in the single-digit nM to sub-nM range, which is two to three orders of magnitude lower than the affinity of hPD-1 for PD-L1. Therefore, based on the receptor occupancy model described above (51), all the antibodies exceeded the affinity threshold of around 20-fold the affinity of hPD-1 for PD-L1/L2.

## 10 PD-1:PD-L1 blockade by anti-PD-1 antibodies

The MOA of anti-PD-1 antibodies is by antagonizing the interaction of hPD-1 with PD-L1/L2. Thus, together with the affinity, the capacity to block the hPD-1:PD-L1 interaction has been a key criterium to select promising anti-PD-1 antibodies for preclinical and eventual clinical development. Several assays have been used to assess the hPD-1:PD-L1 blocking activity including ELISA-based assays (68), cell-based assays with CHO or HEK293 cells stably or transiently modified to express hPD-1 (69), as well as functional inhibition luciferase reporter assays (140). To the best of our knowledge, no standard assay in the field to determine the hPD-1:PD-L1 blocking activity exists nor comparative studies have been published on the blocking activity of the approved antibodies using the same platform and protocol, as the report by Brown et al. (127) for affinity. Therefore, to homogenize as much as possible the comparison of the blocking activity of the anti-PD-1 therapeutic antibodies, we focused on cell-based assays, with CHO or HEK293 cells expressing hPD-1 and competing with PD-L1 and the antibodies in solution for hPD-1 binding. The last column of **Table 4** compiles such information.

As a general trend, the reported hPD-1 blocking activity of the approved therapeutic antibodies falls predominantly within the low nM to sub-nM range. A notable exception is sintilimab, which has an IC_50_ of double-digit nM (132). Nevertheless, even the blocking activity of sintilimab is one to two orders of magnitude lower than that the K_D_ of hPD-1 for PD-L1. Therefore, despite the variability across experimental platforms and protocols, the blocking activity of all the therapeutic anti-PD-1 antibodies with reported blocking activity is consistently stronger than the strength of the hPD-1:PD-L1interaction.

**Figure 6:**
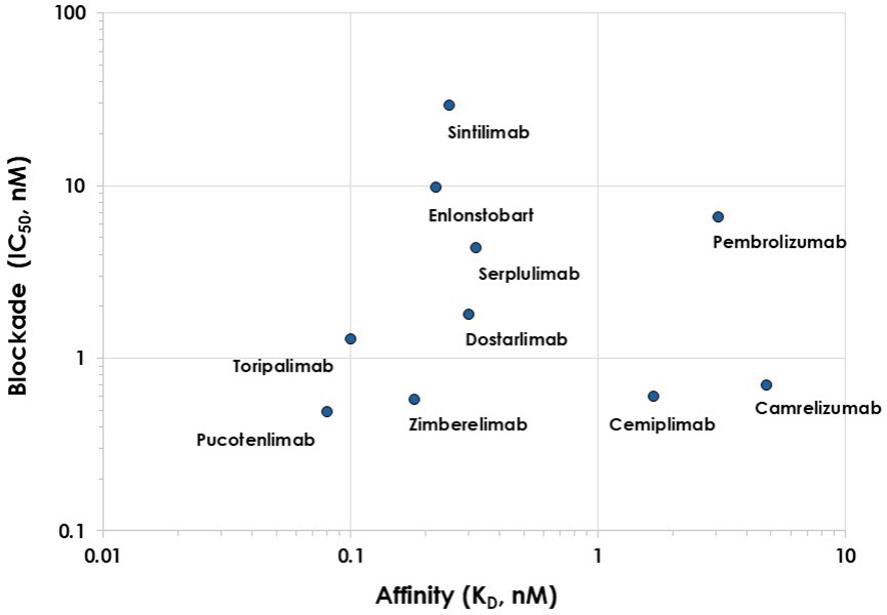
Relationship between K_D_ and IC_50_ determined by the same authors.

A more detailed analysis of the relationship between K_D_ as determined by SPR and IC_50_ values of the blocking activity for 10 of the 16 anti-PD-1 antibodies in which both parameters have been reported by the same authors is shown in **Figure 6**. The binding strength of the anti-PD-1 antibodies does not correlate with hPD-1:PD-L1 blocking activity for the K_D_/IC_50_ pairs. Cemiplimab and camrelizumab have nM affinity but sub-nM blocking activity. In contrast, toripalimab is one of the highest affinity antibodies (0.10 nM) but has nM blocking activity (1.30 nM). Sintilimab, enlonstobart, serplulimab, dostarlimab, and zimberelimab have similar sub-nM affinity (0.18 – 0.32 nM), but the blocking activity spans almost two orders of magnitude, from 0.58 nM (zimberelimab) to 29.20 nM (sintilimab). To visualize the fine details of the mechanism of binding and blocking of the hPD-1:PD-L1 interaction by antibodies we compiled and analyzed all the structures of hPD-1 in complex with antibodies of known structure in the next section.

## 11 Mechanism of binding of anti-PD-1 antibodies

**Figure 7:**
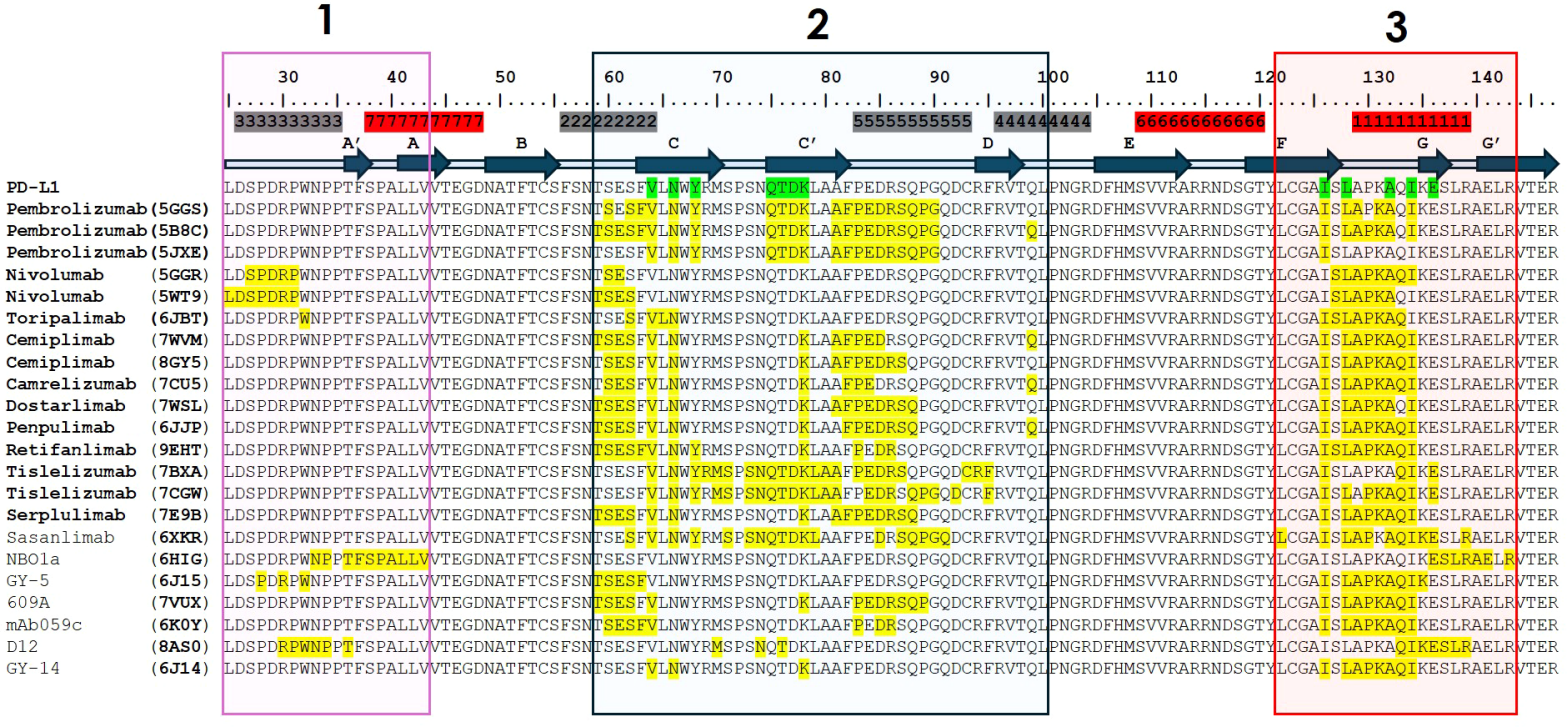
Epitopes of 17 anti-PD-1 antibodies of known crystal structures in complex with hPD-1. Only the sequence of residues 25 to 147 of hPD-1 are shown. On top, the residues identified as antagonist (gray) or agonist (red) epitopes as reported by Suzuki et al., 2023 (141). Blue arrows indicate β-strands and squares indicate the loops connecting them in the hPD-1 secondary structure. The residues in contact with PD-L1 are highlighted in green. The hPD-1 residues in the interface with each antibody were determined using the PDBePISA server (143) and are highlighted in yellow. Boxes (1-3) enclose the epitope regions recognized by the antibodies. Three structures of pembrolizumab, two of nivolumab, two of cemiplimab, and two of tislelizumab solved under different crystallization conditions are included in the analysis to show slight variations in the epitope recognized by the same antibody. PDB IDs are included in parentheses next to each antibody name.

Anti-PD-1 antibodies can be antagonists or agonists depending on where they bind on PD-1. It has been reported (141, 142) that antibodies binding the membrane-proximal extracellular region of PD-1 have agonist activity in sharp contrast with antagonistic antibodies that bind the membrane-distal region of PD-1. **Figure 7** shows a contact map of the residues on hPD-1 involved in the interaction with anti-PD-1 antibodies. The Figure compiles information on 22 structures, with 15 corresponding to approved therapeutic antibodies by different regulatory agencies (**Table 1**). For the latter, three structures of pembrolizumab, two of nivolumab, two of cemiplimab and two of tislelizumab were included in the Figure to gather all the available structural information on these molecules, which have been reported by different laboratories using diverse crystallization conditions and solved at different resolutions. The remaining seven structures correspond to antibodies that have not yet been approved for clinical use. These additional structures allowed us to generalize binding patterns observed in therapeutic antibodies and/or explore mechanisms of binding of anti-PD-1 antibodies still in development.

Overall, three contact regions can be identified on PD-1: (1) the N-loop and β-strands A’ and A (residues 25-43); (2) the C’D loop, and β-strands C and C’ (residues 59-100); and (3) the FG loop (residues 121-144). Most of the antibodies (12 of 17) bind regions 2 and 3, four (nivolumab, GY-5, D12, and toripalimab) bind regions 1, 2 and 3, and one antibody (NBO1a) binds regions 1 and 3.

**Figure 8:**
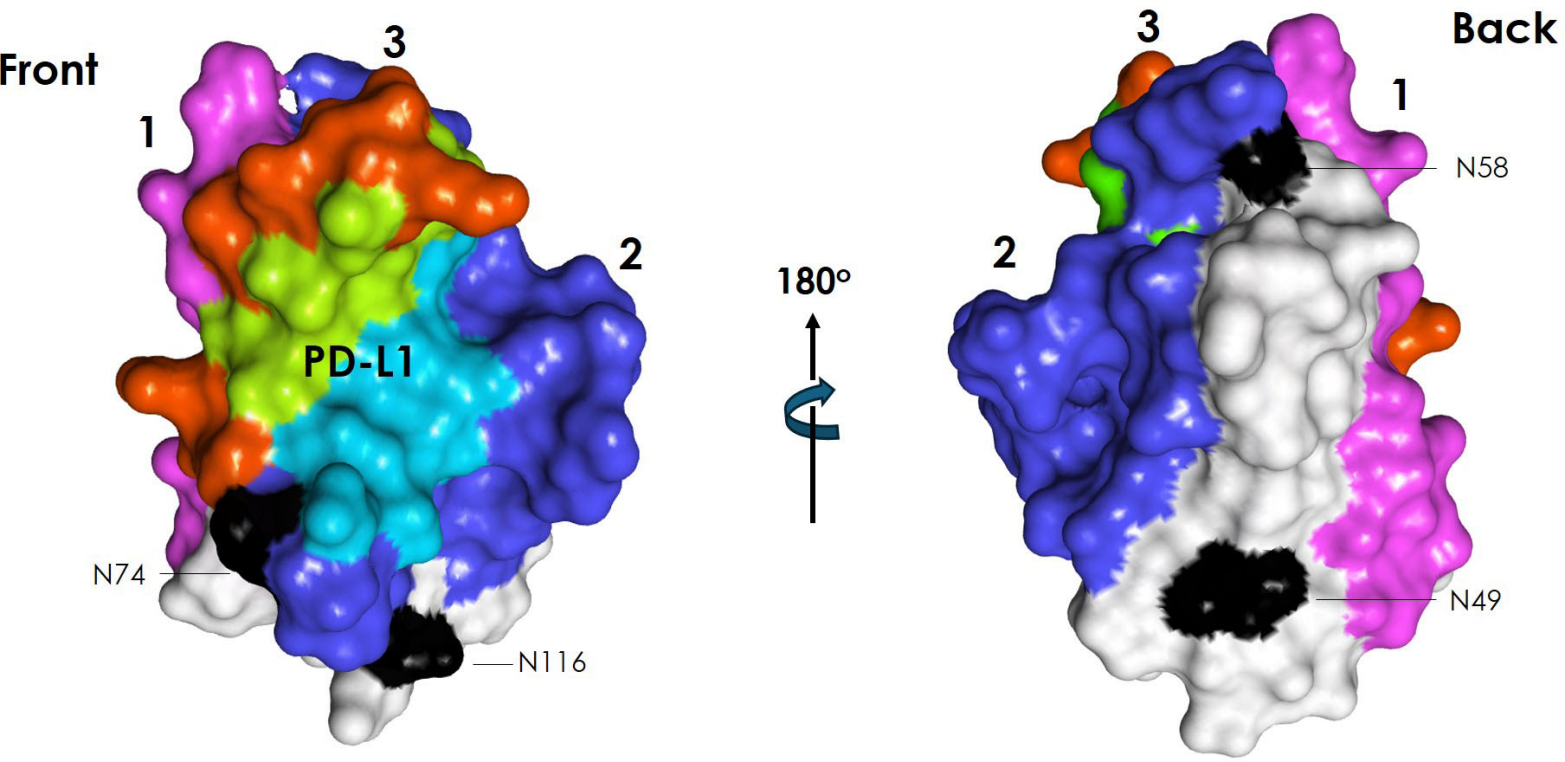
Surface representation of hPD-1 mapping the location of the three antibody binding regions with same color code of **Figure 7**, i.e., region 1(magenta), region 2 (blue), and region 3 (red). Residues of region 3 overlapping with residues involved in PD-L1 binding are highlighted in green. Residues of region 2 overlapping with residues involved in PD-L1 binding are highlighted in light blue. N-glycosylation sites indicated in black. The figure was generated with the PDB ID: 5GGS.

All three contact regions map onto the membrane-distal portion (**Figure 8**) and front face of PD-1, consistent with their antagonist activity (141). The N-glycosylation N58 on the BC loop in region 2, distal from the membrane, is involved in the epitopes recognized by some of the anti-PD-1 antibodies (see below), whereas the remaining three glycosylation sites, N49, N74, and N116, located close to the membrane, do not take part in hPD-1 binding by the antibodies.

As mentioned above, the flexibility of the N-terminal, C’D, BC and FG loops at the membrane-distal region of PD-1 plays an important role in the interaction with the antibodies. For instance, the crystal structure of nivolumab bound to PD-1 is the only one in which the N-terminal extension is visible, indicating that nivolumab stabilizes the flexible N-terminal region (58). Other hPD-1 structures discussed below in complex with antibodies have shown that upon antibody binding, the C’D, BC and FG loops adopt conformations that add contacts to the PD-1:antibody interface and hence, strengthen the interaction with hPD-1 (144).

**Figure 9:**
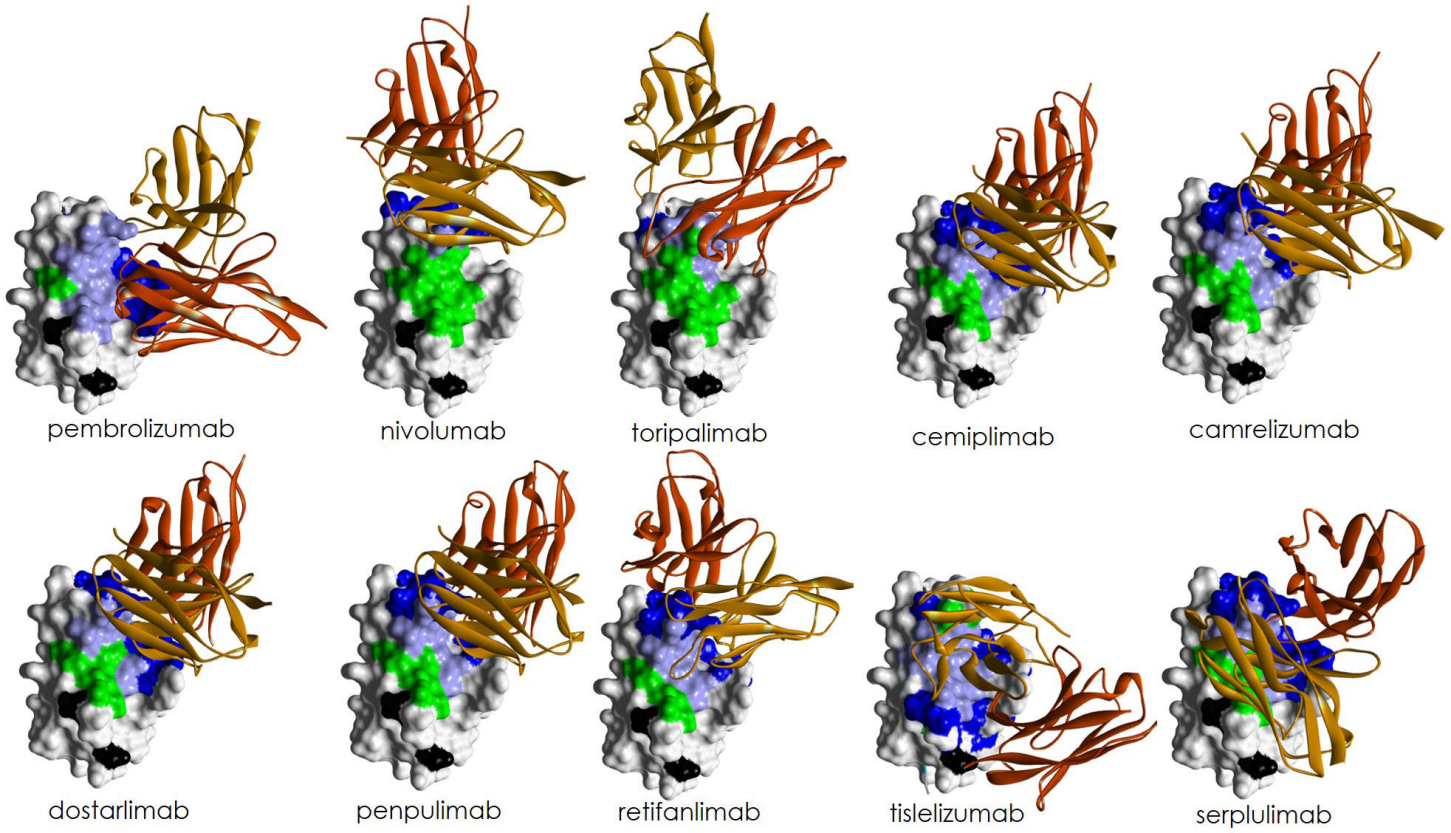
Surface representation of hPD-1 in complex with the V regions (ribbon representation) of diverse anti-PD-1 antibodies. The hPD-1 of the complexes were aligned using as reference the PDB ID: 5GGS to show the structures in the same orientation with respect to hPD-1. The color code is as follows: V_H_: dark brown; V_L_: light brown. On the hPD-1 surface, contact residues of PD-L1 on hPD-1: green; contact residues of the antibody on hPD-1 but not overlapping with contact residues of PD-L1 on hPD-1: dark blue; contact residues of the antibody on hPD-1 overlapping with binding residues of PD-L1 on hPD-1: light blue; N-glycosylation sites: black. The Figure of each complex was generated with the coordinated listed in **Table 2**, replacing hPD-1 of each PDB file with the coordinates of hPD-1 of the PDB ID: 5GGS to homogenize the view of the complexes.

Nivolumab and pembrolizumab bind to hPD-1 with similar nM affinity. Their epitopes overlap in region 3 and share two residues in region 2. However, binding of nivolumab to the N-terminal located in region 1 of PD-1 does not participate in the binding of PD-L1 to hPD-1, implying a different orientation of the antibody with respect to hPD-1 (**Figure 9**) that leads to a significant difference in the overlapping surface of pembrolizumab and nivolumab with PD-L1 binding site. Pembrolizumab’s epitope covers a large part of the interface with the PD-L1 binding site whereas nivolumab does cover a much less portion of it. It has been proposed (145) that the difference in orientation of these antibodies with respect to hPD-1 and the surface covered by their interaction could contribute to differences observed in *in vitro* hPD-1 blockade by these antibodies (145).

Toripalimab binds hPD-1 with sub-nM affinity as measured by SPR (0.10 nM) (128) but has a blocking activity closer to nivolumab than pembrolizumab. Toripalimab’s long HCDR3 of 16 amino acids and the long LCDR1 create a cavity in the antibody binding site that embraces the FG loop. The long HCDR3 contributes with multiple hydrogen bonds (60) to the hPD-1:toripalimab interface, with the long LCDR1 contacting only one residue of the N-terminal region. This interaction resembles the relative orientation of nivolumab with respect to hPD-1.

Cemiplimab, camrelizumab, dostarlimab, penpulimab and to a lesser extent retifanlimab bind hPD-1 with a similar V_H_ and V_L_ orientation. These antibodies have short HCDR3 loops mainly binding hPD-1 with V_H_, whereas V_L_ serves as the predominant region to compete with binding of PD-L1 (72). Cemiplimab and camrelizumab have affinities nM range and sub-nM blocking activities, similar to that of pembrolizumab. However, cemiplimab and camrelizumab bind the N58 glycan on the BC loop of hPD-1, as indicated by a substantial decrease in binding affinity in N58-glycan-deficient hPD-1 (146). Further, by using combinations of protein- and cell-based assays, it has been shown that the blocking activity of cemiplimab and camrelizumab is sensitive to variations in fucose (146). As sPD-1 competes with cell-bound hPD-1 for binding to the antibodies, fucosylation may lead to distinct binding patterns and functional activity of these anti-PD-1 antibodies (146).

The N58 glycosylation site also contributes to penpulimab binding to PD-1 (61). However, comparing the glycosylated hPD-1 with a solved complex previously reported (57) that lacked the glycan, it was shown that the glycosylation had no effect on the overall structure or conformational change of hPD-1. Therefore, hPD-1 glycosylation of N58 is likely involved in the penpulimab binding by direct interaction, and not via induction of conformational changes. Structural analysis (71) and sequence alignment also indicate that cemiplimab binds to N58 glycan chains with conserved HCDR2 regions similar to camrelizumab and penpulimab. However, clinical studies for cemiplimab do not report a high prevalence of capillary hemangiomas as observed for camrelizumab (58.6%) (147). This indicates that although the conserved N58 glycan promotes the binding of these two antibodies to hPD-1 in a similar mode, the binding specificity may vary due to the type of contacts in the CDR (see sequence alignment in **Figure 5** for differences in the CDR sequences) of the antibodies responsible for binding hPD-1.

Unlike the other anti-PD-1 therapeutic antibodies, the epitope of tislelizumab covers almost entirely (80%) the PD-L1 binding site (68), providing complete blockade of the interaction PD-1:PD-L1. Binding of tislelizumab includes CC’ loop of PD-1 and avoids the flexible N-terminal, C’D and FG loop regions encompassed in binding of the other anti-PD-1 antibodies. Tislelizumab has high binding affinity (0.11 nM) mostly driven by a slower dissociation rate than the other anti-PD-1 antibodies (68), enabling a more sustained target binding, which may contribute to its functional activity (68).

Serplulimab binds hPD-1 with nM K_D_ similar to nivolumab and pembrolizumab. It also exhibits similar performance *in vitro* and *in vivo* studies than nivolumab (69). However, it does not bind region 1 and has a different orientation with respect to hPD-1 than nivolumab. Serplulimab has shown enhanced tumor killing activity in specific preclinical combination settings compared to pembrolizumab upon co-administration with anti-TIGIT or anti-LAG3 inhibitors. Mechanistically, serplulimab combinations effectively reduce tumor microenvironment Treg cell populations, augment effector and memory T cell populations, and modulate with higher potency genes associated with diverse facets of the immune system, surpassing the effects of the combination with pembrolizumab (148).

Among the structures of hPD-1 in complex with antibodies that have not been approved for clinical use, NBO1a represents a unique case of study. It binds region 1 and the C-terminal residues of region 3. None of the interactions between NBO1a and hPD-1 overlap with the residues involved in the interaction between hPD-1 and PD-L1 (59). NBO1a recognizes hPD-1 via the opposing face of the PD-L1 interaction and does not block the PD-1:PD-L1 interaction. Interestingly, NBO1a epitope does not overlap with that of pembrolizumab either but has shown comparable antitumor activity in preclinical models. Combination therapy using both pembrolizumab and NBO1a enhanced tumor suppression in an immunogenic mouse tumor model (59), suggesting that NBO1a acts predominantly through the CD28 coreceptor.

Another case of study recently reported on an anti-PD-1 antibody (149), indicates that it binds in region 2 as a typical antagonist antibody but also recognizes an amino acid stretch between regions 2 and 3, where no other anti-PD-1 antibody of known structure binds hPD-1. This antibody, named UDIZ-007, does block the PD-1/PD-L1 interaction in binding and cell-based assays. At a dose of 10 mg/kg every three days for a total of six intraperitoneal administrations eradicated MC38-hPD-L1 colon tumors in B-hPD-1 transgenic mice expressing hPD-1 at day 17, with no relapse until the end of the study at day 56, as reported in the original study (149). Thus, UDIZ-007´s mechanism of binding may not be by directly outcompeting hPD-L1 interaction with hPD-1 but instead by steric hindrance and/or by binding to the C’D loop of hPD-1 and stabilizing a conformation that prevents the interaction with hPD-L1.

In summary, anti-PD-1 antibodies clinically approved for treatment of cancer all bind the front face and the membrane distal region of hPD-1 but the fine details of the interaction with hPD-1 indicate diverse mechanisms of binding. The flexibility of the N-terminal region, and C’D, BC and FG loops of hPD-1 play an important role in the mechanism of binding of some antibodies. The glycan at position N58 of hPD-1 affects the interaction with others. One of the therapeutic antibodies approved for clinical use, serplulimab, has shown enhanced tumor killing activity upon co-administration with anti-TIGIT or anti-LAG3 inhibitors. Another antibody that has not yet been approved for clinical use, NBO1a, which recognizes an epitope that does not overlap with the PD-L1 binding site nor with the epitope recognized by pembrolizumab, combined with the latter, has been reported to enhance antitumor activity over pembrolizumab in specific experimental settings. Therefore, combinations of anti-PD-1 antibodies with antibodies targeting diverse oncology relevant molecules or targeting hPD-1 with distinct mechanism of binding could lead to differences in efficacy and/or enhance antitumor activity over monotherapy.

## 12 PD-1–based bispecific antibodies

Besides therapeutic combinations with monospecific anti-PD-1 antibodies, a new generation of molecules has emerged in which an anti-PD-1 antibody or fragment is linked to other modules targeting oncology related receptors or ligands into a single molecular entity. Unlike combination therapy using two independent monospecific antibodies, the bi- or multi-specific format enables simultaneous target engagement with shared pharmacokinetics and a defined molecular stoichiometry. Currently, two bispecific antibodies targeting hPD-1 have received regulatory approval by NMPA: cadonilimab (AK104) and ivonescimab (AK112).

Cadonilimab targets hPD-1 and CTLA-4, also known as a cis-targeting bispecific antibody (150). CTLA-4 largely controls early lymphoid T-cell activation and promotes regulatory T-cell suppression, whereas hPD-1 suppresses effector T-cell activation in the peripheral setting while maintaining inhibitory features in the tumor microenvironment (151, 152). Cadonilimab was approved for the treatment of recurrent or metastatic cervical cancer following platinum-based chemotherapy in 2022, demonstrating clinically relevant activity associated with immune-related adverse events compatible with simultaneous checkpoint blockade (153).

**Figure 10:**
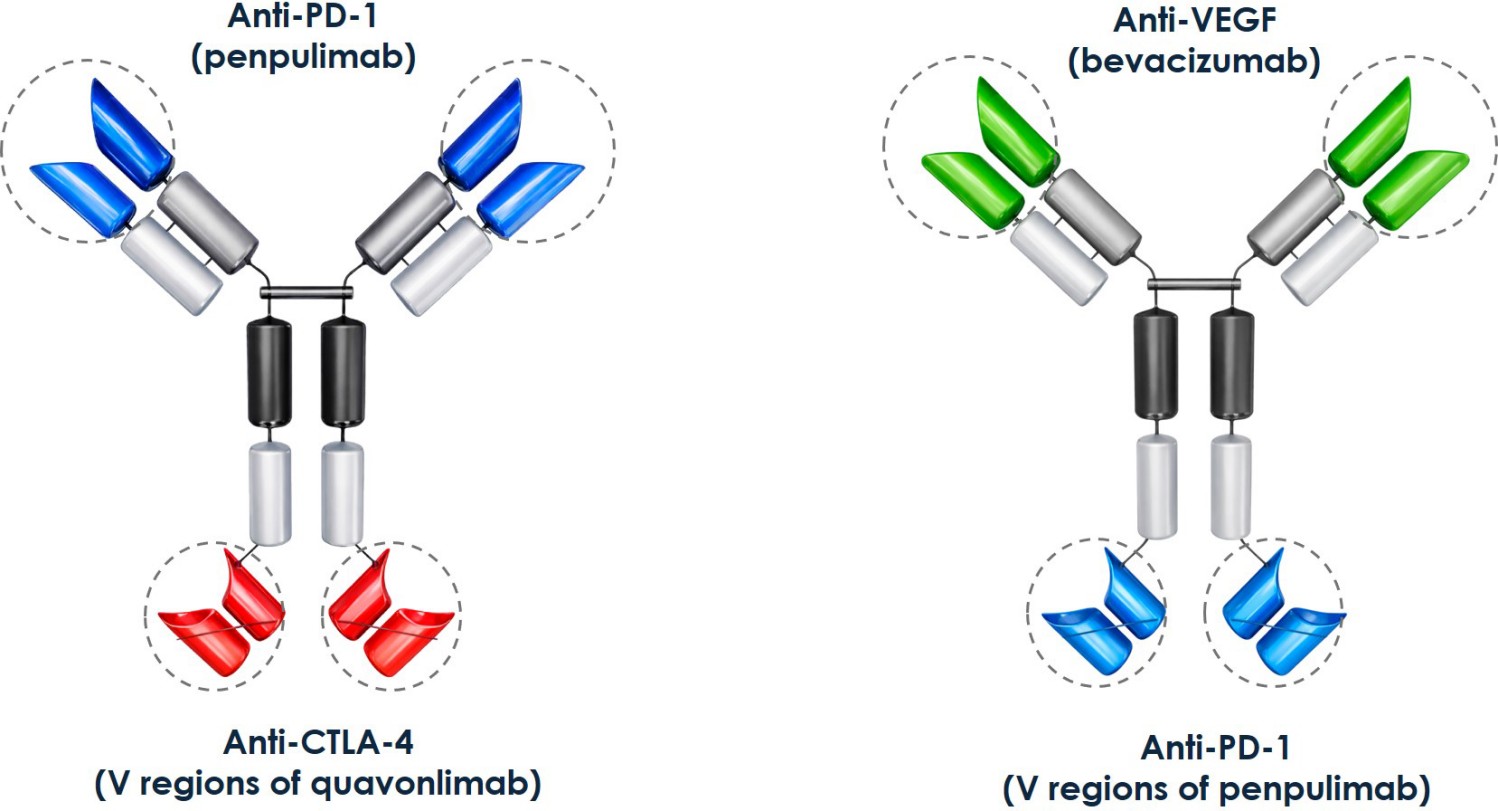
Structural organization of approved hPD-1–based tetravalent bispecific antibodies. **(A)** Cadonilimab (AK104), a tetravalent (2 + 2) IgG-like bispecific antibody containing two hPD-1–binding domains and two CTLA-4–binding domains assembled within a single IgG-based framework. **(B)** Ivonescimab (AK112), a tetravalent (2 + 2) IgG-like bispecific antibody engineered with two hPD-1–binding domains and two VEGF-binding domains.

At the C-terminal of penpulimab (anti-PD-1 antibody), cadonilimab links via a standard (G_4_S) linker two scFvs of quavonlimab (anti-CTLA-4) (**Figure 10**) (154) in a tetravalent (2 + 2) IgG-like bispecific format. The Fc region has LALA mutations plus G237A (154). As discussed above, these mutations minimize the effector functions of the molecule without affecting the half-life. Interestingly, in addition to the typical N-linked glycosylation site of the IgGs at Asn298, cadonilimab has an additional N-glycosylation site at Asn524, which corresponds to position 55 of the HCDR2 (Kabat numbering) of quavonlimab, despite that antibodies with glycosylation sites other than the site at Asn298 tend to be deprioritized during the selection process of the candidates for further development.

Affinity of cadonilimab using Fortebio Octet (154) has been determined in 0.04 nM with a high-density hPD-1 and CTLA-4 coated sensor (50 nM), whereas the K_D_ with a low-density coated sensor (10 nM) resulted in a value of 0.48 nM. The K_D_ of penpulimab measured by these authors (154) was 0.20 and 0.36 nM in both high- and low-density sensors, respectively, which are close to the K_D_ measured by SPR (0.59 nM) – see **Table 4**. Thus, the 10-fold difference of cadonilimab at high density with respect to low density sensor chip seems to be an avidity effect, which could facilitate cadonilimab retention in areas of the cells with high density of PD-1 and CTLA-4 (154).

Ivonescimab targets hPD-1 and VEGF-A. It was designed to combine T-cell activation with blockade of VEGF-A signaling. Aside from being involved in angiogenesis, VEGF is known to play a role in immune suppression through the impaired maturation of dendritic cells, constraining T-cell trafficking, and supporting immunosuppressive cells inside tumors (155, 156). By antagonizing hPD-1 and binding VEGF-A simultaneously, ivonescimab restores T-cell activity in the tumor microenvironment. Ivonescimab was approved by NMPA for advanced EGFR-mutant non–small cell lung cancer following treatment with EGFR tyrosine kinase inhibitors (157). Compared with pembrolizumab, ivonescimab significantly improved median progression-Free Survival (PFS) to 11.1 from 5.8 months with pembrolizumab (158).

Ivonescimab is built with the scFv of penpulimab linked to the C-terminal of the anti-VEGF antibody bevacizumab (**Figure 10**). Like cadonilimab, ivonescimab has an IgG1 isotype engineered with the LALA mutations, but it does not have the G237A mutation. The binding kinetics of ivonescimab to recombinant human PD-1 and VEGF as determined by Fortebio Octet analysis yielded K_D_ values of 0.25 nM for hPD-1 and 0.33 nM for VEGF. These affinity values compare well with those of penpulimab (0.20 – 0.36 nM, see above) and are slightly higher than the K_D_ of bevacizumab (0.66 nM). Further, blocking of the hPD-1 signaling (IC_50_ = 22.54 nM) and VEGF signaling (IC_50_ = 3.13 nM) is comparable to the parental antibodies, penpulimab and bevacizumab.

Finally, it should be noted that although cadonilimab and ivonescimab maintain an IgG-like structure that could facilitate manufacturability and predictability in pharmacokinetics, reconciling affinity and target occupancy, minimizing the immune-related toxicity, and preserving the good biophysical properties of the parental antibodies, continue to pose outstanding development hurdles for developing bispecific molecules. Nevertheless, cadonilimab and ivonescimab have demonstrated two plausible extensions of the hPD-1 blockade by combining dual immune checkpoint inhibition or adding the benefit of a linkage to blocking a key growth factor that stimulates angiogenesis. Therefore, these innovative anti-PD-1-based drugs that reuse elements from already approved monospecific therapeutic antibodies including penpulimab (targeting hPD-1), quavonlimab (targeting CTLA-4), and bevacizumab (targeting VEGF), have opened the door to more sophisticated and efficacious therapies to treat cancer.

## 13 Conclusions

Targeting hPD-1 with antibodies has been a paradigm-shift in cancer treatment. Currently, 16 therapeutic anti-PD-1 antibodies and two bispecific molecules have globally been approved for clinical use. Although these antibody-based drugs have been developed using different discovery and engineering methods, and have been characterized by diverse assays, instrumentation and experimental protocols, some general trends that could serve as a reference for developing new anti-PD-1 therapeutic antibodies can be highlighted.

Most of the clinically approved anti-PD-1 antibodies and the two bispecific molecules have humanized V regions, with only six out of 18 being fully human. The V regions of these antibodies have in common FRs or entire human germline genes with favorable developability profiles, which have recurrently been used in therapeutic antibodies. IgG4 has been the isotype of choice to engineer the vast majority of the anti-PD-1 antibodies, with only two antibodies and the bispecific molecules having IgG1 isotype with LALA mutations that abrogate the effector functions. The reported immunogenicity data suggest a slight trend toward higher ADA incidence in humanized compared to fully human anti-PD-1 antibodies. However, this trend should be interpreted with caution due to differences in methodologies to assess ADAs, sampling strategies, patient populations, terminology and strategies for collecting, analyzing, and presenting immunogenicity results.

From a functional viewpoint, although quantitative differences among K_D_ values have been reported by different laboratories for the same antibody, the affinity for hPD-1 falls in the single-digit nM to sub-nM range. With this affinity range, antagonizing the hPD-1:PD-L1 interaction has led to a blocking activity within the low nM to sub-nM range across experimental platforms. This blocking activity is two to three orders of magnitude stronger than the affinity of hPD-1 for PD-L1 and PD-L2, which is in double-digit nM or low µM range.

All the antibodies bind the front face and the membrane distal region of hPD-1. Despite these commonalities, the fine details of the interaction with hPD-1 indicate distinct mechanisms of binding. In some antibodies, the flexibility of the N-terminal, C’D, BC and FG loops of hPD-1 play an important role in the binding mechanism. In others, the glycan at position N58 is involved in binding and affects the interaction with the antibodies, resulting in distinct binding patterns depending upon the glycosylation of hPD-1. Importantly, combination therapies of anti-PD-1 antibodies with distinct modes of hPD-1 binding have been reported to enhance antitumor activity in specific experimental settings. Further, one of the two bispecific antibodies approved for human therapy, has shown superior clinical efficacy as compared to the therapeutic antibodies used to engineer the bispecific molecule, paving the way to the development of more efficacious cancer treatments targeting hPD-1.
